# Miltefosine regulates gene expression related to morphogenesis and zinc metabolism in *Candida albicans* and *Candida tropicalis*

**DOI:** 10.1128/spectrum.00519-26

**Published:** 2026-08-26

**Authors:** Murilo Moreira dos Santos, Vinícius de Morais Barroso, Sofia Prampero, Márcia Cristina Furlaneto, Milton Yutaka Nishiyama-Jr, Kelly Ishida

**Affiliations:** 1 Laboratory of Antifungal Chemotherapy, Department of Microbiology, Institute of Biomedical Sciences, University of São Paulo, São Paulo, Brazil; 2 Laboratory of Applied Toxinology, Butantan Institute, São Paulo, Brazil; 3 Department of Microbiology, Center of Biological Sciences, State University of Londrina, Paraná, Brazil; University of Guelph, Guelph, Ontario, Canada

**Keywords:** zincophore, zinc, filamentation, morphogenesis, miltefosine, *Candida tropicalis*, *Candida albicans*

## Abstract

*Candida albicans* and *Candida tropicalis* are major hyphae-forming pathogens causing candidiasis. The rise of antifungal resistance underscores the need for new therapeutic strategies. Miltefosine (MFS), an alkylphosphocholine designated as an orphan drug for invasive candidiasis, has shown inhibitory, fungicidal, and antibiofilm effects against *Candida* spp. In this study, we used microbiological and transcriptomic approaches to investigate the mechanisms underlying inhibition of growth and filamentation by MFS in *C. albicans* and *C. tropicalis*. The minimum inhibitory concentrations of MFS for all isolates ranged from 1 to 4 µg/mL, and antibiofilm effects were observed at 2–8 µg/mL (biofilm formation), and at 16–128 µg/mL (mature biofilm). MFS impaired yeast adhesion on polystyrene, inhibited the yeast-to-hyphae transition on different filament-inducing media, and induced alterations in the chitin and chitosan exposure in the cell wall. The inhibitory effect on filamentation promoted by MFS can be explained, in part, by the negative regulation of gene expression associated with virulence and transcriptional factors, such as *ECE1*, *HWP1*, *ALS3*, *EFG1*, *RAS1*, *CYR1*, *TEC1*, *NGT1*, and *CEK1* for *C. albicans* and *ALS1*, *SAP3*, *EFG1*, *BCR1*, and *NGT1* for *C. tropicalis*. In addition, the RNA-seq and qPCR analyses highlighted the most enriched upregulated genes in response to MFS involved in zinc metabolism, especially zincophore *PRA1* and zinc transporters *ZRT1* and Z*RT2*, and improved fungal growth was observed under MFS treatment in zinc-supplemented media. Our data provided further evidence of the mode of action of MFS in inhibiting growth and filamentation, particularly concerning the involvement of zinc metabolism in MFS-induced stress.

**IMPORTANCE:**

Miltefosine is a repurposed antifungal agent that effectively targets molds and yeasts, including *Candida albicans* and *Candida tropicalis*. By integrating phenotypic and transcriptomic approaches, we demonstrate that miltefosine exerts fungicidal activity while broadly impairing key virulence attributes, including adhesion, biofilm development, and yeast-to-hyphae transition. The transcriptional profiling revealed upregulation of filamentation-blockers and zinc machinery genes, and downregulation of virulence factor genes alongside modulation of cell wall component exposure. RNA-seq further highlighted a massive response involving zinc homeostasis, particularly the zincophore and zinc transporters, suggesting metal metabolism as part of the drug’s mechanism of action. Given the limited antifungal arsenal and the clinical burden of invasive candidiasis, these findings expand the mechanistic framework underlying miltefosine activity and reinforce its repositioning as a candidate for antifungal therapy in two highly relevant fungal pathogens. Collectively, this work advances our understanding of antivirulence strategies and supports drug repurposing to address antifungal resistance.

## INTRODUCTION

The *Candida* genus is the most prevalent pathogen in mucosal and systemic infections (1). Invasive candidiasis and candidemia are responsible for about 1.5 million people affected annually and a high mortality rate of ~65% (2). Around 50% of cases are caused by *Candida albicans*, which is globally distributed, while *Candida tropicalis* is the second most frequent species in countries from Latin America, Africa, and Asia (3–5). In addition, both species are associated with the emergence of antifungal resistance (6, 7).

*C. albicans* and *C. tropicalis* were classified by the World Health Organization as fungal pathogens of critical and high priority, respectively, rising in this scenario of medical mycology together with *Candidozyma auris* (formerly *Candida auris*), *Nakaseomyces glabratus* (formerly *C. glabrata*), and *Candida parapsilosis* (8). The higher prevalence of both species in hospitalized and immunosuppressed patients is due to their presence in the skin and mucosa microbiota, their ability to transit from yeast to pseudohyphae/hyphae, and to form biofilms that are considered the main virulence attributes (7).

Biofilms are defined as microbial communities capable of adhering to biological or non-biological surfaces through adhesins and extracellular matrix production (9). They are the most resilient and resistant way in which *Candida* presents itself to the environment or to the host. The presence of an extracellular matrix, efflux pump overexpression, and persister cells are some factors that make biofilm cells resistant to components from the host immune system and antifungal agents when compared with their counterpart planktonic cells (10). The complex biofilm regulatory network comprises a wide variety of components expressed during this lifestyle, including morphological transitions that enable filamentous forms to penetrate the host tissues (9). In this scenario, it is important to highlight the lack of therapeutic options to treat invasive infections caused by *Candida* species and relatives, which are limited to only three drug classes (azoles, echinocandins, and polyenes); furthermore, only echinocandins are reference antifungals for treating candidemia associated with biofilms (11, 12).

In the last decade, there have been advances in the development of drugs with improved pharmacokinetics, reduction of toxicity, and novel mechanisms of action, contributing to the expansion of the antifungals pipeline (13). The newest molecules approved by the Food and Drug Administration (FDA) and other health agencies are rezafungin, fosmanogepix, ibrexafungerp, oteseconazole, and others, which are recently approved or in the clinical and preclinical phases (14). In addition, the drug repurposing approach is an expanding field of research that has contributed to antifungal development. Miltefosine (MFS), an alkylphospholipid used for the treatment of breast cancer and leishmaniasis, has been widely investigated as a new antifungal agent by our research group, and it was designated by the FDA as an orphan drug for invasive candidiasis (15) based on several studies *in vitro* and *in vivo* against pathogenic fungi (16).

Previous studies showed the broad-spectrum action of MFS against yeast and filamentous fungi and its fungicidal effect (16, 17). In *C. albicans* and *C. tropicalis*, MFS inhibited fungal growth and biofilm formation on catheter surfaces and other materials, with a potent filamentation inhibition (18–20). Furthermore, MFS reduced *C. albicans* infection and inhibited hyphae formation in the *Galleria mellonella* larvae model and in a murine model of oral and vaginal candidiasis (19, 21, 22).

Nevertheless, some knowledge gaps keep persisting, such as how MFS inhibits filamentation, biofilm formation, and other virulence factors in *C. albicans* and *C. tropicalis*. The transition from yeast to hyphal form is a complex process regulated by a wide genetic network. Filamentation is a virulence factor strongly correlated with increased pathogenesis. Both *C. tropicalis* and *C. albicans* share key characteristics, making the yeast-to-hyphae transition a major focus in studies aiming to inhibit virulence (23). Therefore, here we investigated the molecular mechanisms of MFS action on the growth, filamentation, and biofilm formation of *C. albicans* and *C. tropicalis* through transcriptomic profiling.

## MATERIALS AND METHODS

### Fungal collection

Five *Candida albicans* isolates (SC5314 or ATCC MYA-2876 and four clinical isolates from candidemia) and six *Candida tropicalis* isolates (ATCC 200956 and five clinical isolates from candidemia [3] and oropharyngeal [2]) were previously identified as susceptible to fluconazole (FLC-S, 3 *C. albicans* and 3 *C. tropicalis*) and resistant to fluconazole (FLC-R, 2 *C. albicans* and 3 *C. tropicalis*) (24, 25).

The strains were obtained from the fungal collections of the Laboratory of Antifungal Chemotherapy (ICB/USP, São Paulo/SP) and the Furlaneto Lab (CCB/UEL, Londrina/PR) and were stored in Brain and Heart Infusion broth (BHI, Difco, USA) with 20% glycerol at −80°C. The yeasts were recovered on Sabouraud Dextrose Agar (SDA, Difco, USA), and purity was confirmed on CHROMagar *Candida* (Difco, USA). Prior to experiments, a new 24 h culture of *C. albicans* and *C. tropicalis* was carried out on SDA at 35°C.

### Antifungal

MFS was purchased from Cayman Chemical Co. (Ann Arbor, MI) and was dissolved in sterile ultrapure water to obtain a working solution, which was stored at −20°C. Fluconazole (FLC, Sigma-Aldrich) was used as a standard antifungal, and it was dissolved in dimethyl sulfoxide (DMSO, Sigma-Aldrich) to obtain a stock solution stored at −20°C.

### Antifungal susceptibility testing

The MFS *in vitro* inhibitory activity was evaluated using the broth microdilution method M27-A4 (26), and the minimum inhibitory concentration (MIC) was defined as the lowest MFS concentration inhibiting 90% of fungal growth due to the lack of inhibition 50% values, a profile commonly seen for MFS. Additionally, the minimum fungicidal concentration (MFC) was determined after culturing each fungal inoculum treated at concentrations ≥MIC on free-drug SDA for 24 h at 35°C. MFC was defined as the lowest concentration that killed >99.9% of the initial inoculum. The effect was considered fungicidal when the MFC was equal to or less than 4× MIC value; otherwise, the effect was considered fungistatic (27). *Candida parapsilosis* ATCC 22019 was used as the quality control strain, and fluconazole was used as a standard antifungal. The modal FLC MIC value for *C. parapsilosis* was 1 µg/mL, targeting the expected data according to CLSI documents; thus, we may assume that the experiment was conducted correctly, and the results are reliable.

### Growth kinetics of *Candida tropicalis* in zinc-supplemented media

Cultures grown in Yeast Potato Dextrose broth (YPD, BD, USA) at 35°C for 24 h were harvested, washed with sterile phosphate-buffered saline (PBS) pH 7.0, and adjusted to a final concentration of 1 × 10³ colony-forming units (CFU)/mL. Aliquots of the standardized inoculum were transferred to wells containing YPD medium free or supplemented with ZnSO₄·7H₂O at a final concentration of 9 μM and MFS at final concentrations of 0.5, 1, or 2 μg/mL. Untreated cells grown in only YPD supplemented or not with zinc were used as controls in 96-well microplates. The growth curves were incubated at 35°C in a microplate reader (Biotek Epoch 2, USA) with orbital shaking, and optical density measurements were recorded every 30 min for 24 h. Growth curves were generated from the absorbance values obtained throughout the incubation period and compared between the different treatment conditions.

In addition, we also evaluated the *C. tropicalis* growth curves using the zinc-specific media, a zinc-supplemented and defined medium adapted from (28) consisting of glucose (8 g/L), (NH₄)₂SO₄ (8 g/L), KH₂PO₄ (3.6 g/L), Na₂HPO₄·12H₂O (1.2 g/L), MgSO₄·7H₂O (0.2 g/L), and D-biotin (0.1 μg/L) with or without ZnSO₄·7H₂O (9 μM). Growth curves were performed in biological triplicate and experimental quadruplicate, with the final data plotted as the mean of all values obtained.

### Dose-response assay

The dose-dependent effect of MFS on *C. tropicalis* growth was evaluated using a broth microdilution-based approach. Briefly, fungal cells were harvested from 24 h cultures in YPD media at 35°C, washed with PBS, and adjusted to 1 × 10³ CFU/mL in YPD broth. Aliquots of 100 µL of the standardized inoculum were dispensed into 96-well microplates containing 100 µL of YPD broth supplemented or not with zinc and MFS at final concentrations of 4, 3.5, 3, 2.5, 2, 1.5, 1, and 0.5 µg/mL. The microplate was incubated at 35°C for 24 h, and the experiment was performed in biological triplicate.

### Miltefosine effect on adhesion and biofilm

#### Anti-adhesion assay

Yeasts at 2 × 10^6^ CFU/mL were treated with MFS at MIC values (2 μg/mL for *C. albicans* and 4 μg/mL for *C. tropicalis*) in PBS in a 24-well polystyrene plate (Kasvi, Brazil) for 1 h at 35°C. After incubation, the supernatant was removed, the wells were washed once with PBS, and the adhered cells were fixed with 4% formaldehyde in PBS for 1 h. Then, the fixative was discarded, the cells were washed once with PBS, and stained with 0.2% crystal violet (Sigma-Aldrich) for 5 min and washed twice with PBS. Adhered cells were counted in 25 random fields using a 40× objective lens, and the adhesion inhibition percentage was calculated relative to untreated yeasts.

#### Antibiofilm assay

An aliquot of 100 μL of yeasts at 1 × 10^6^ CFU/mL, in Roswell Park Memorial Institute 1640 medium (RPMI-1640) buffered with 0.165 M 3-(N-morpholino)propanesulfonic acid (RPMI-MOPS, pH 7.0) (both from Sigma-Aldrich), was dispensed into the wells of a 96-well flat-bottomed polystyrene plate (Kasvi, Brazil) and incubated at 35°C for 1.5 h, under shaking (150 rpm). For biofilm formation, non-adhered cells were removed, and RPMI-MOPS medium was added and incubated for 24 h at 35°C, under shaking.

For the evaluation of MFS on biofilm formation, after the adherence period, the supernatant was discarded, and 100 μL of different concentrations of MFS diluted in RPMI-MOPS were dispensed into each well, and the microplate was incubated for 24 h at 35°C, under shaking (150 rpm). For the evaluation of MFS on mature biofilms, 24 h preformed biofilms were treated with MFS in RPMI-MOPS for 24 h at 35°C under shaking (150 rpm). Then, the total biofilm biomass was quantified by crystal violet stain (Sigma-Aldrich), the metabolic activity of sessile cells from biofilm cells was determined by the tetrazolium salt reduction assay (XTT and Menadione, Sigma-Aldrich), and cell viability was evaluated by CFU counts and by fluorescence microscopy using the FUN-1 marker (Thermo Fisher, USA) (29, 30).

### Miltefosine effect on morphogenesis

*Candida albicans* (SC5314) and *Candida tropicalis* (IAL-01) were used as representative isolates of each species due to a greater and clearer filamentation phenotype previously observed on Spider agar. The same samples were applied for all subsequent experiments. Yeasts were treated with concentrations ranging from 0.125 to 16 μg/mL of MFS for 24 h at 35°C in different hyphae-inducing media to determine the MIC values. The hyphae-inducing media included RPMI-MOPS medium, liquid and solidified Spider medium, liquid Lee medium, and SM liquid medium supplemented with N-acetylglucosamine (SM-GlcNAc) (31–36). In addition to MIC value determination, cell images were captured under a fluorescence microscope (EVOS FL, Thermo Fisher Scientific) after staining with Calcofluor White M2R (1 mg/mL, Sigma-Aldrich). In solidified Spider medium containing a sub-MIC concentration of MFS, 10 μL of fungal suspension (1 × 10^3^ CFU/mL) was inoculated, incubated for a week at 35°C for colony observation under a 10× magnifying lens, and the images captured with the aid of a 12-megapixel camera with a focal aperture of 1.5 (EVOS FL, Thermo Fisher Scientific).

### Miltefosine effect on the cell wall structural components

The yeasts of *C. albicans* SC5314 and *C. tropicalis* IAL-01 (1 × 10^7^ CFU/mL), untreated and treated with the MIC value of MFS in RPMI-MOPS medium, were grown for 24 h at 35°C, without shaking, for the obtention of only yeasts in the treated samples. After incubation, the inoculum was centrifuged, washed twice with PBS, and standardized at a cell density of 1 × 10^6^ CFU/mL and fixed in 4% formaldehyde in PBS for 1 h at room temperature. Then, the cells were stained using different fluorescent markers: eosin Y disodium (1 mg/mL) for chitosan, Calcofluor White 2R (1 mg/mL) for chitin, Aniline Blue (5 mg/mL) for β 1,3-glucan, and Concanavalin A-Alexa Fluor 488 (1 mg/mL) for mannan (37, 38). After 10 min of incubation at room temperature and in the dark, cells were washed in PBS, and subsequently, the fluorescence intensity was determined for 100,000 events by flow cytometry using a BD Accuri C6 (BD, New Jersey, USA).

### Miltefosine effect on the expression of virulence genes

Primer sequences used in this assay are listed in Table S1, based on the literature or designed by the current authors using the *Candida* Genome database (http://www.candidagenome.org) and Primer3Plus (https://www.primer3plus.com). For *C. albicans* SC5314, the following genes were analyzed: *SAP1*, *SAP6*, *ALS3*, *HWP1*, *CDR1*, *ERG11*, *ECE1*, *RIM8*, *CEK1*, *RAS1*, *CPH1*, *CPH2*, *TEC1*, *NGT1*, *CYR1*, and *EFG1*. For *C. tropicalis* IAL-01, the following genes were used: *SAP3*, *ALS1*, *ALS3*, *HWP1*, *CDR1*, *ERG11*, *BCR1*, *WOR1*, *NGT1*, *EFG1*, *PRA1*, *ZRT1*, and *ZRT2. ACT1* was used as the reference (housekeeping) gene for both species.

Yeasts at 1 × 10^7^ CFU/mL, untreated and treated with MFS (6 μg/mL, which allowed only yeast form), were grown in RPMI-MOPS medium for 24 h at 35°C, without shaking. After incubation, cells were washed in PBS and collected for total RNA extraction with the RNeasy Plant Mini kit (Qiagen, Hilden) and quantified using a Nanodrop (Thermo Fisher, Massachusetts). RNA quality was considered acceptable with a 260/280 nm ratio between 2 and 2.2. Subsequently, the genomic DNA was digested with the DNase I kit (Invitrogen, USA), and cDNA synthesis was performed using the High-Capacity cDNA Reverse Transcription Kit (Thermo Fisher, USA). The qPCR reaction was performed using the SYBR Green qPCR Master Mix Low Rox kit (Nova Biotecnologia, Brazil). Gene expression was calculated using the relative quantification by the 2^−ΔΔCT^ method.

### Transcriptome of *Candida tropicalis* yeasts treated with miltefosine

#### Samples obtention

Standardized yeast inoculum (1 × 10^6^ CFU/mL) of *C. tropicalis* IAL-01 was treated or not with 5 µg/mL MFS in 10 mL of RPMI-MOPS at 35°C for 20 h, without shaking. These growth conditions ensured inhibition of filamentation and sufficient material for RNA sequencing analysis. Then, the samples were centrifuged, washed with PBS, and the cells were disrupted with a micropestle in liquid nitrogen. The total RNA was obtained using the RNeasy Plant Mini kit (Qiagen, Hilden), and the DNase I kit (ThermoFisher, USA) was used to remove residual genomic DNA. Then, the samples were maintained at −20°C until sent for RNA sequencing.

#### Samples PCA analysis

Principal component analysis (PCA) was performed to evaluate the global expression profile and replicate consistency among untreated and MFS-treated *C. tropicalis* samples. The analysis was conducted using the 3,000 highly variable genes, a normalized expression matrix generated for visualization purposes, after log₂(x + 1) transformation and removal of genes with zero variance across samples. Genes were used as variables and samples as observations, and PCA was calculated using the *prcomp* function in R. The first two principal components were plotted with ggplot2, with samples colored according to experimental condition: untreated samples (C1_S32 and C2_S33) and MFS-treated samples (T1_S34 and T2_S35). PCA was used only as an exploratory visualization approach to assess sample distribution and was not used for differential expression testing.

#### Data processing and analysis

The RNA-sequencing included the validation of four samples per bioanalyzer; construction of RNA library by poly A enrichment using the Illumina Stranded mRNA kit; quantification by quantitative qPCR-based methods, clustering, and sequencing (NextSeq 2 × 100 bp, average coverage of 24 million paired reads per sample). For demultiplexing and separation of fastq files for each sample used for library preparation, the raw paired-end reads from NextSeq showing Phred quality-based (Q) ≥ 30 were processed by the CASAVA library (version 1.8.2). An in-house pipeline was used for pre-processing of raw fastq files, including filtering for contaminants, such as PhiX and UniVec databases (https://ftp.ncbi.nlm.nih.gov/pub/UniVec/), using the aligner Bowtie 2, version 2.2.5 (39), with the parameters of --very-sensitive-local and -k 1. In the quality control, reads were filtered by quality, minimum read length of 40 bp, poly A/T/N tails, and removal of adapter/primer sequences, using the program Trimmomatic version 0.39 (40) with the following parameters: ILLUMINACLIP:NextGenPrimerAdaptersUniVec.fa:2:30:10 LEADING:5 TRAILING:5 SLIDINGWINDOW:25:25 MINLEN:40 HEADCROP:0. After the data preprocessing step, high-quality paired reads were mapped to the reference genome *C. tropicalis* MYA-3404 with the Hisat2 alignment program (41), based on 6,258 genes. For the Hisat2 software, the defined parameters were a minimum intron size of 30 pb, suppress unpaired alignments (--no-mixed), suppress discordant alignments (--no-discordant), and retain only uniquely mapped reads to keep only the best mapped paired-end reads for further quantification analyses. For gene abundance analysis, the featureCounts tool from the Bioconductor Rsubread package (42) was used with the following parameters: GTF.featureType = “exon,” GTF.attrType = “gene_id,” isPairedEnd = TRUE, requireBothEndsMapped = TRUE, minOverlap = value 1, allowMultiOverlap = FALSE, countMultiMappingReads = FALSE. The raw count matrix generated by featureCounts was used as the input for all downstream differential expression analyses performed with DESeq2. For exploratory analyses, expression abundance estimation, visualization, clustering analyses, and heatmap generation, normalized expression estimates were additionally calculated using counts per million (CPM), fragments per kilobase of exon per million mapped fragments, and transcripts per million. Then, the RNA sequences were deposited in the NCBI database under the number PRJNA1400329.

The identification of differentially expressed genes (DEGs) was performed using the R Bioconductor package DESeq2 (v1.22.2) (43). Differential expression analyses were conducted exclusively using the raw count matrix generated by featureCounts, following the standard DESeq2 workflow and its internal median-of-ratios normalization procedure. Genes with CPM > 1 in at least two replicates were retained for differential expression analysis. DEGs were defined using a conservative criterion combining Benjamini-Hochberg-adjusted *P* value ≤ 0.001 and fold change ≥ 3 (corresponding to |log₂FoldChange| ≥ 1.58). This stringent cutoff was applied to prioritize genes with robust expression changes for downstream enrichment analyses and mechanistic interpretation of the MFS response. CPM filtering was applied only as a low-expression filtering criterion prior to downstream analyses for DESeq2 statistical modeling. StringDB and KEGG were used for enrichment analyses.

We analyzed the 1,733 DEGs from *C. tropicalis* untreated (C1_S32, C2_S33) and MFS-treated (T1_S34, T2_S35) samples, resulting in 975 unique genes after UniProt annotation, removing rows with non-finite values or zero variance across samples. For clustering and heatmap visualization analyses only, normalized expression matrices derived from CPM-transformed expression values were used. Between-sample normalization was performed using quantile normalization using limma’s normalizeBetweenArrays method and applying log2(x + 1) transformation. This normalization strategy was applied exclusively for visualization, exploratory transcriptomic pattern analyses, and clustering procedures. To enrich the informative structure, the genes were ranked by median absolute deviation across samples and then median-centered each gene (subtracting its sample median) to emphasize relative profiles.

Hierarchical clustering was conducted on the centered matrix using Spearman rank-correlation converted to distance (1–ρ) with complete linkage for both genes and samples; followed by Optimal Leaf Ordering (R package seriation) was used to refine leaf order without altering original clustering dendrogram topology. Gene partitions were defined by cutting the gene dendrogram at 55% of total tree height, and heatmaps were generated with pheatmap (green–white–red palette, 75 levels) using the precomputed row and column dendrograms, omitting row names for readability, and displaying a row side-color bar encoding cluster membership; for each partition, we exported a submatrix with columns ordered by the sample dendrogram. All analyses were performed in R-statistics (≥4.2) with pheatmap, limma, and optionally seriation.

### Statistical analyses

The statistical analyses were performed using the GraphPad Prism 8.0 program, and *P* values < 0.05 were considered statistically significant.

## RESULTS

### Miltefosine has fungicidal effect and inhibits all biofilm development phases of *Candida albicans* and *Candida tropicalis*

All *C. albicans* and *C. tropicalis* isolates were inhibited by MFS at 1–4 µg/mL, including FLC-R strains, and it showed fungicidal action at 2–8 µg/mL (Table 1). Here, we also evaluated the effect of MFS in all steps of biofilm formation. In the adhesion phase on polystyrene, MFS at MIC values inhibited all isolates with a significant reduction in the number of adhered yeasts when compared to untreated yeasts (*P* < 0.0001). For *C. albicans*, the reduction in adhesion ranged from 19% to 37%, while for *C. tropicalis*, the reduction ranged from 17% to 75% (Table 1). Additionally, all biofilms were inhibited by MFS, which was most effective in inhibiting the biofilm formation at 2–8 µg/mL, while in the mature phase, MFS inhibited the sessile cells only at 16–128 µg/mL (Table 1).

**TABLE 1:** Effect of MFS in the growth of planktonic cells, adhesion on polystyrene surface, and biofilms (formation and pre-formed) of *Candida albicans* and *Candida tropicalis* clinical isolates

Strains	Planktonic cells^*a*^ (µg/mL)		Biofilm cells^*b*^ (µg/mL)		Adhesion^*c*^ (mean ± standard deviation)	
	MIC	MFC	BIC formation	BIC pre-formed	No drug	MFS (inhibition %)
*C. albicans*						
IAL-40^R*e*^	2	2	8	16	2,405.5 ± 92.5	1,521 ± 90.4 (37%)^*d*^
IAL-42^R*e*^	2	2	4	16	2,187.5 ± 71.2	1,484.8 ± 67.8 (32%)^*d*^
IAL-51	2	2	4	16	1,577.3 ± 66.3	1,119.3 ± 54.5 (29%)^*d*^
IAL-53	2	2	4	16	2,152.3 ± 116.4	1,733.8 ± 54.6 (19%)^*d*^
SC5314	2	2	4	32	2,013.3 ± 114.4	1,387 ± 52.2 (31%)^*d*^
*C. tropicalis*						
ATCC 200956^R*e*^	1	2	2	32	738.55 ± 84.82	465.6 ± 89.38 (37%)^*d*^
IAL-04^R*e*^	4	8	4	64	514.72 ± 81.54	381.4 ± 51.19 (26%)^*d*^
IAL-08^R*e*^	4	8	8	32	511.1 ± 76	423.6 ± 58.54 (17%)^*d*^
IAL-01	4	8	8	64	1,155.75 ± 117.53	290.1 ± 48.85 (75%)^*d*^
49.07	2	4	4	32	557.56 ± 69.98	388.86 ± 28.52 (30%)^*d*^
335.07	2	4	8	128	549.6 ± 44.27	285.1 ± 38.88 (48%)^*d*^

^*a*^ MIC (minimum inhibitory concentration) is the lowest concentration that inhibits 90% of fungal growth. MFC (minimum fungicidal concentration) is the lowest concentration that kills >99.9% of the initial fungal inoculum. MIC and MFC are modal values obtained from experiments, in biological triplicate.

^*b*^ BIC (biofilm inhibitory concentration) is the modal value of the lowest concentration that inhibits 90% of the metabolic activity of biofilm cells by XTT reduction assay, in experimental triplicate and biological triplicate.

^*c*^ MFS (2 μg/mL for *C. albicans* and 4 μg/mL for *C. tropicalis*) inhibits significantly yeast adhesion by counting randomly 25 fields, in biological triplicate. Values in parentheses are the percentage of adhesion inhibition.

^*d*^ *P<0.0001 when compared with untreated yeasts (no drug) (Student’s *t*-test).

^*e*^ R, resistant to fluconazole (24). ATCC 200956 is resistant to amphotericin B (24). All strains were isolated from candidemia of the human patients, except 49.07 and 335.07 strains, that were isolated from oropharyngeal human patients.

**Fig 1:**
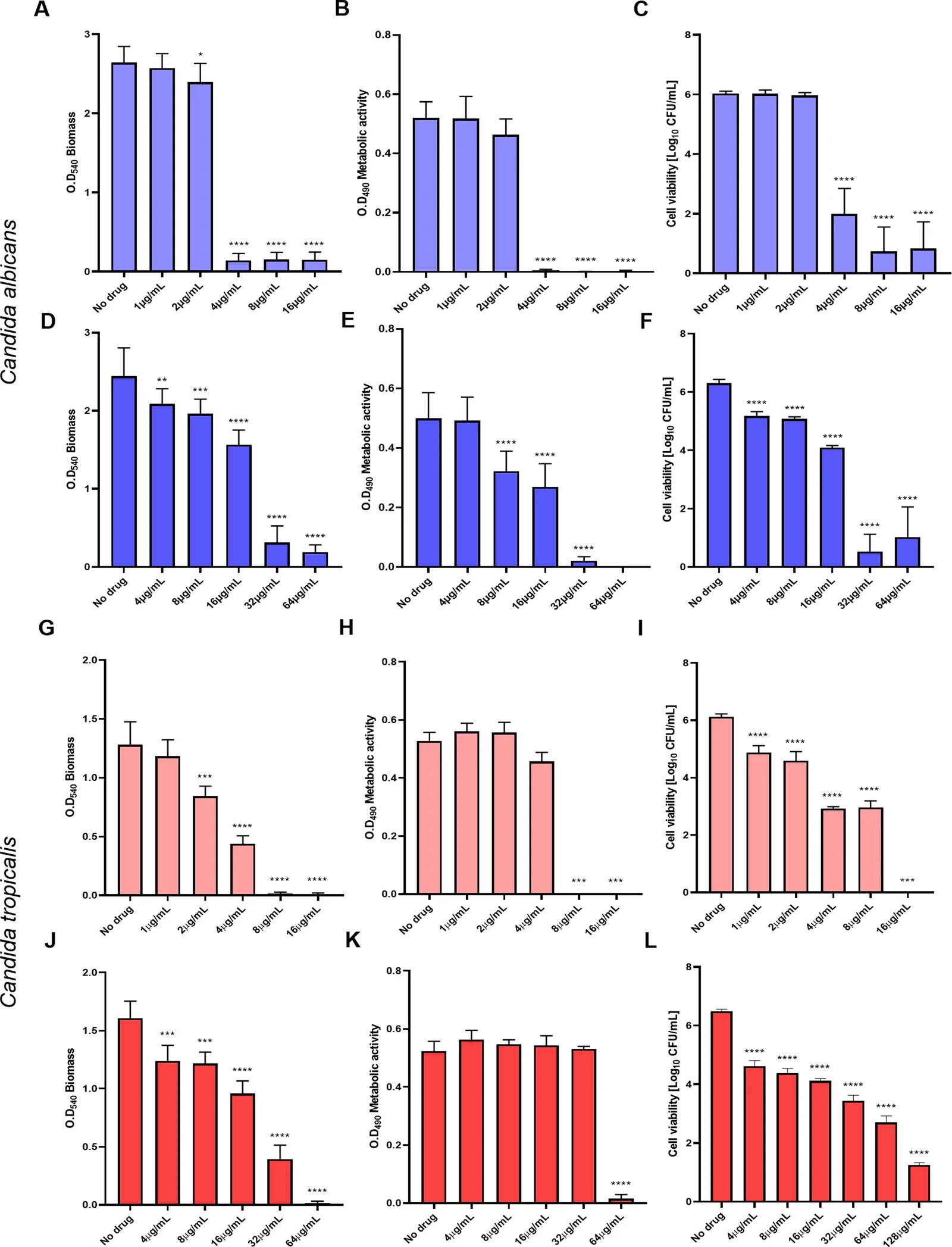
Miltefosine can eradicate *Candida* biofilms. Biofilms of *Candida albicans* SC5314 and *Candida tropicalis* IAL-01, in formation (**A–C and G–I**) and pre-formed (**D–F and J–L**), treated or not with miltefosine were evaluated by determining the total biofilm biomass through crystal violet staining (A, D, G, and J), metabolic activity by XTT reduction assay (B, E, H, and K), and cell viability by CFU count (C, F, I, and L). *P < 0.05, **P < 0.01, ***P < 0.001, and ****P < 0.0001 when compared to untreated cells (no drug), in technical quadruplicate and biological triplicate (one-way ANOVA with Dunnett post-test).

The determination of total biomass, metabolic activity, and viability of cells from biofilms was carried out using the same representative strain of *C. albicans* (SC5314) and *C. tropicalis* (IAL-01) under MFS treatment (Fig. 1). MFS at concentrations ≥2 µg/mL significantly reduced the total biomass, metabolism, and CFU in treated cells during biofilm formation, reaching complete eradication at higher concentrations of 8–16 µg/mL (Fig. 1A through C and G through I). Additionally, as expected, MFS showed activity on pre-formed biofilms at higher concentrations of 32–128 µg/mL (≥80% inhibition) compared with its effect on the biofilm formation, although a significant reduction was observed in the biomass, metabolic activity, and cell viability at ≥4 µg/mL of MFS (Fig. 1D through F and J through L). It is important to highlight that the metabolic activity of cells from *C. albicans* biofilms was more affected by MFS than *C. tropicalis* biofilms, in both biofilm development phases (Fig. 1).

Furthermore, we captured images of the biofilms untreated and treated with MFS using light and fluorescence microscopies (Fig. S1 through S4). FUN-1 can be metabolized by viable cells, resulting in the formation of vacuolar structures that exhibit red fluorescence. During biofilm formation, we observed dead cells of *C. albicans* at 2–4 µg/mL, whereas *C. tropicalis* showed cell death at 8–16 µg/mL (Fig. S1 and S3). In mature biofilms, cell death in *C. albicans* was observed at 64–128 µg/mL, and in *C. tropicalis* at 128 µg/mL (Fig. S2 through S4). Therefore, a reduction in live cells after treatment was observed with increasing MFS concentrations, corroborating the CFU counting in Fig. 1.

MFS also reduced hyphae/pseudohyphae formation in both filament-competent species observed in light and fluorescence (Calcofluor White) microscopy (Fig. S1 through S4), as well as described previously by other authors using scanning electron and fluorescence microscopies (18, 20). Thus, in this work, we explored the molecular mechanisms underlying the inhibition of fungal growth, biofilm formation, and morphogenesis induced by MFS.

### Miltefosine inhibits yeast-to-hyphae transition of *Candida albicans* and *Candida tropicalis* in different filamentation-inducing media

The inhibition of growth and yeast-to-hyphae transition by MFS was evaluated using the following filamentation-inducing media: RPMI-MOPS (multiple pathways), Lee (*CPH2* and *TEC1* pathways), SM-GlcNAc (*NGT1* pathway), and Spider (*EFG1* for liquid media and *CPH1* for solid media) (32, 34, 35). The macromorphological and micromorphological images were taken using representative strains of *C. albicans* (SC5314) and *C. tropicalis* (IAL-01), although the same morphologies were observed in all tested strains.

**Fig 2:**
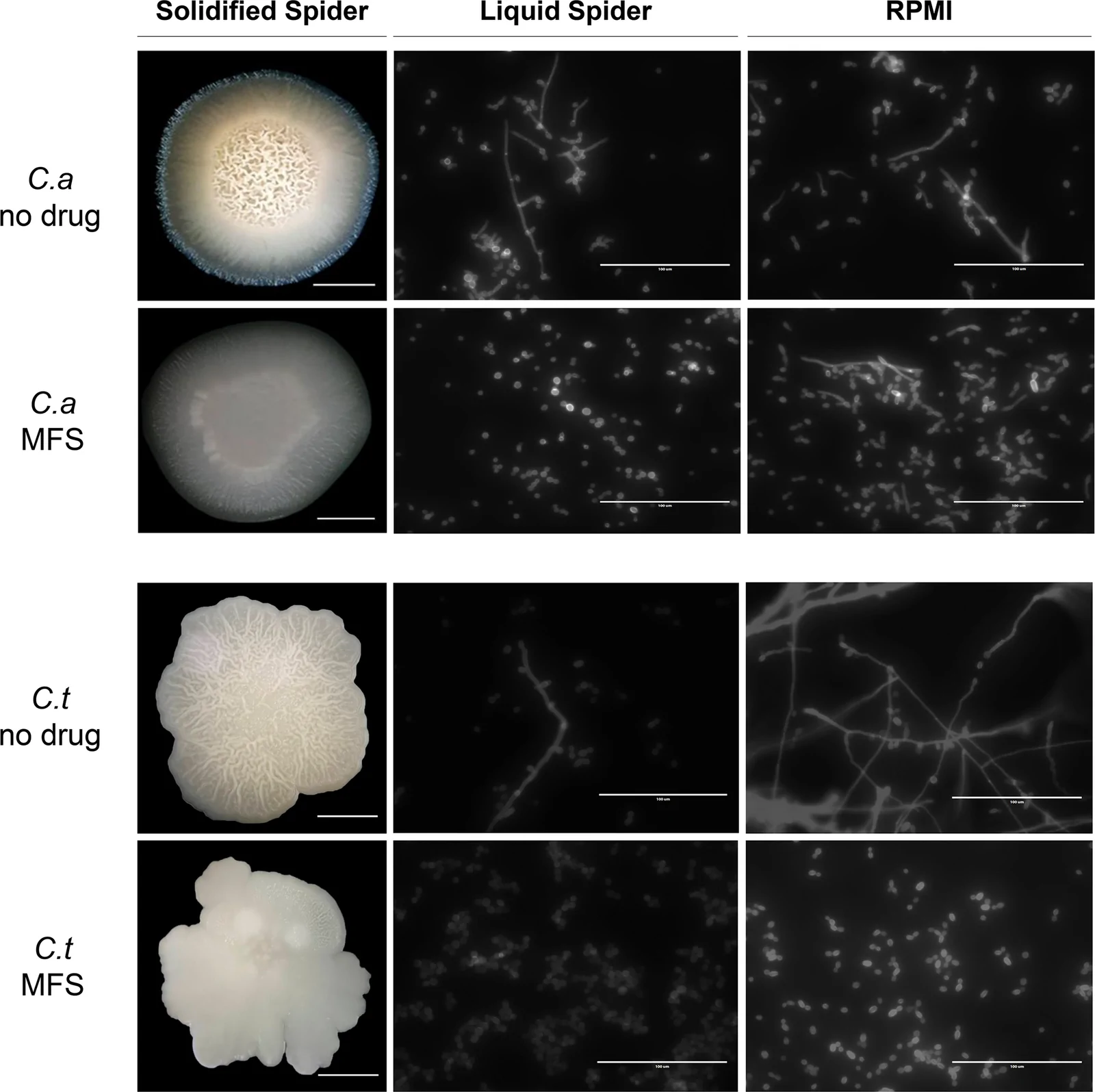
Miltefosine can block *Candida albicans* and *Candida tropicalis* filamentation across different filament-inducing media. Each row represents *C. albicans* in the untreated condition (no drug), *C. albicans* treated with MFS, *C. tropicalis* in the untreated condition (no drug), and *C. tropicalis* treated with MFS, respectively. Colony images represent solidified Spider medium supplemented with MFS at 1 µg/mL for *C. albicans* and 2 µg/mL for *C. tropicalis*. Bars = 4 mm for colony images and bars = 100 µm for fluorescence microscopy images.

The susceptibility profile of isolates to MFS was maintained when grown in filamentation-inducing media (Table S2). MFS at sub-MIC in solidified Spider medium partially inhibited the filamentation of *C. albicans* and *C. tropicalis* due to the presence of filamentous structures in the colony peripheral region, especially in *C. albicans* (Fig. 2). In all liquid filamentation-inducing conditions, the total inhibition of hyphae and pseudohyphae by MFS was observed, except for *C. albicans* in RPMI-MOPS and SM-GlcNAc media, where a reduction in the number and complexity of hyphae/pseudohyphae, in terms of branching and length, was observed (Fig. 2 and Fig. S5). These data may suggest that MFS inhibits hyphae/pseudohyphae formation, but total inhibition may fail when filamentation is induced by contact, as seen in Spider agar.

### Miltefosine alters chitin and chitosan exposure in *Candida albicans* and *Candida tropicalis* cell walls

**Fig 3:**
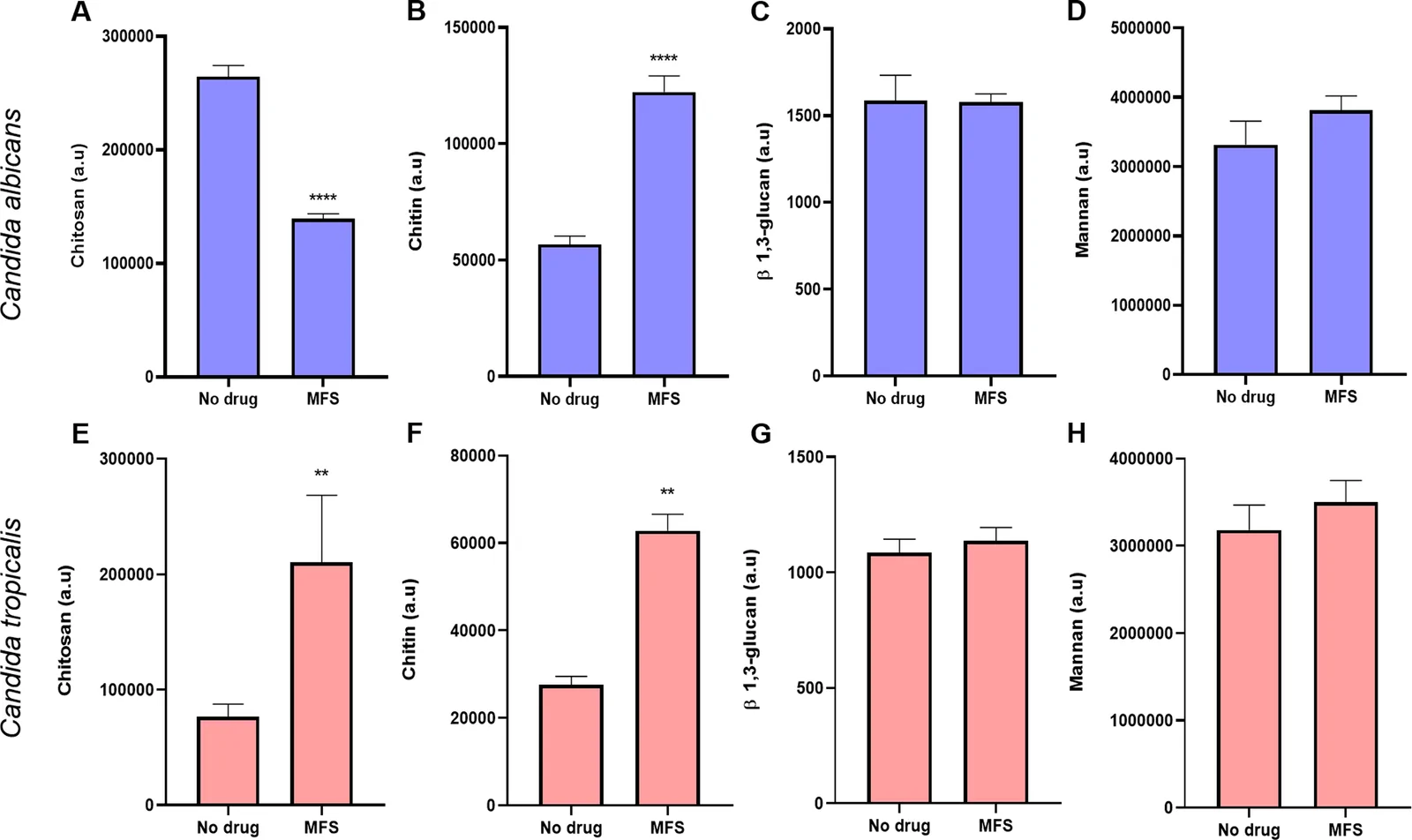
Miltefosine alters cell wall structural component exposure of *Candida*. Yeasts of *Candida albicans* SC5314 (**A–D**) and *Candida tropicalis* IAL-01 (**E–H**) were treated or not (no drug) with MFS at MIC value for 24 h at 35°C and were labeled for quantification of the cell wall components exposure in a flow cytometer using the following fluorescent markers: eosin Y disodium (1 mg/mL for 10 min) for chitosan (**A and E**), calcofluor white (1 mg/mL for 10 min) for chitin (**B and F**), aniline blue (5 mg/mL for 10 min) for β 1,3-glucan (**C and G**), and concanavalin A conjugated with Alexa Fluor 488 (1 mg/mL for 10 min) for mannoproteins (**D and H**). **P < 0.01 and ****P < 0.0001 when compared with no drug group (Student’s *t*-test), in technical and biological triplicates. a.u., arbitrary unit.

The fluorescence quantification of yeasts treated or not with MFS in RPMI-MOPS medium labeled with eosin Y disodium for chitosan, calcofluor white for chitin, aniline blue for β 1,3-glucan, and concanavalin A conjugated with Alexa Fluor 488 for mannan may indicate how the cell wall remodels under culture conditions. MFS significantly induced a reduction in the chitosan exposure and an increase in the chitin exposure in the cell wall of *C. albicans* (Fig. 3A and B). On the other hand, in *C. tropicalis*, there was a significant increase in the chitosan and chitin exposure (Fig. 3E and F). For β 1,3-glucans and mannoproteins, no significant changes were observed (Fig. 3).

### Miltefosine downregulates genes associated with morphogenesis and other virulence factors of *Candida albicans* and *Candida tropicalis*

**Fig 4:**
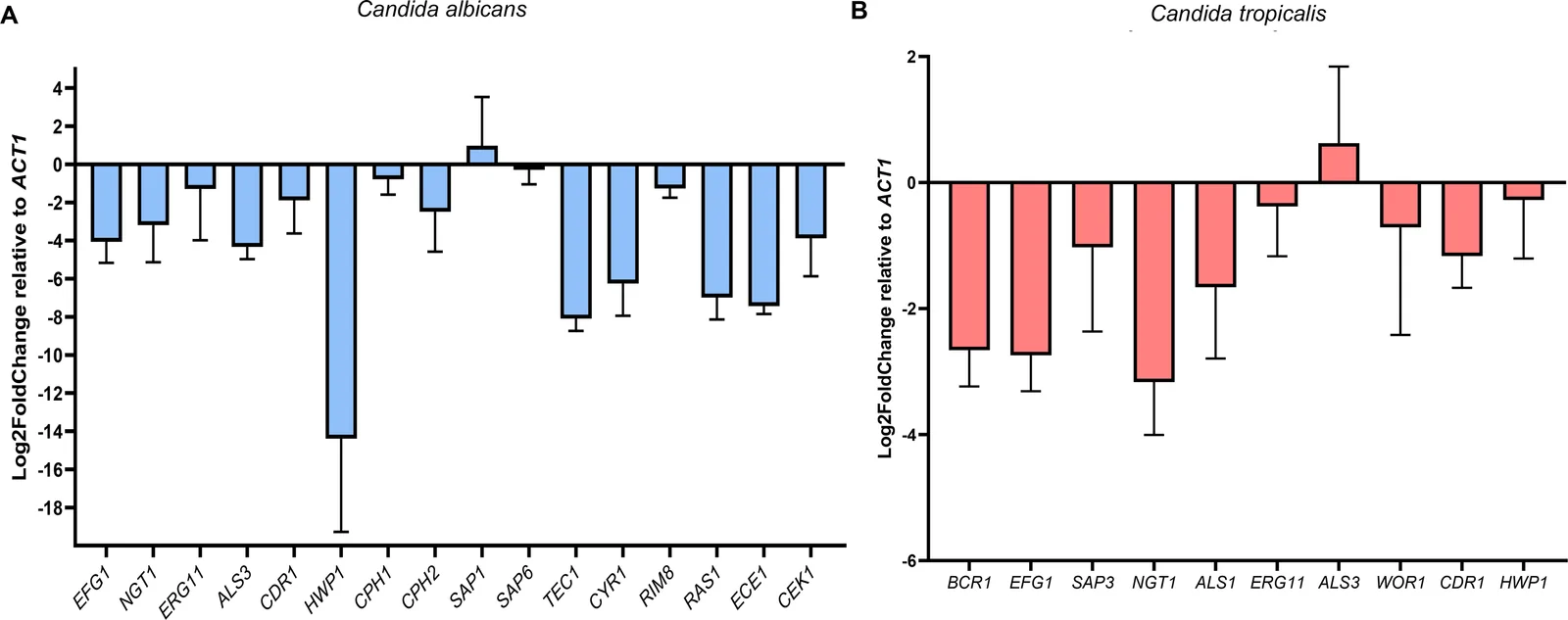
Miltefosine downregulates genes associated with virulence factors in *Candida*. Relative expression of virulence factors in cells from *Candida albicans* SC5314 (**A**) and *Candida tropicalis* IAL-01 (**B**) under exposure to miltefosine at an inhibitory concentration compared to untreated cells in RPMI-MOPS at 35°C for 24 h. qPCR reaction was performed to quantify gene expression levels during MFS treatment. Data were normalized with the beta-actin gene (*ACT1*). The assay was performed in biological triplicate and technical triplicate.

After treatment with MFS for 24 h at 35°C in RPMI-MOPS, *C. albicans* SC5314 cells were collected to determine the expression of virulence genes: *HWP1*, *ALS3*, *SAP1*, *SAP6*, *ECE1*, *RIM8*, *CEK1*, *RAS1*, *CPH1*, *CPH2*, *TEC1*, *NGT*, *CYR1*, and *EFG1*. We observed a decrease in Log_2_FC ≥ 2 in the gene expression of adhesins (*HWP1* and *ALS3*), candidalysin (*ECE1*), GlcNAc-specific transporter (*NGT1*), and filamentation regulators (*EFG1*, *TEC1*, *CYR1*, *RAS1*, and *CEK1*) in MFS-treated cells (Fig. 4A). Other genes, *RIM8*, *ERG11*, *SAP1*, *SAP6*, *CPH1*, and *CPH2*, showed minimally altered expression (less than twice compared with untreated yeasts), and one of them is associated with signaling pathways for filamentation by contact (*CPH1* gene) (32), corroborating the partial inhibition of filamentation observed on Spider agar.

Fewer genes, focused on the primary virulence factors described for the species, were tested for *C. tropicalis* (23). After the same treatment condition, we observed a more prominent decrease in the gene expression of *BCR1*, *EFG1*, and *NGT1*. At the same time, *SAP3*, *ALS1*, and *CDR1* were slightly downregulated. No relevant change was observed for *ERG11*, *WOR1*, *ALS3*, and *HWP1*. In fact, a major downregulation profile can be observed under MFS treatment, highlighting the morphogenesis-related gene *EFG1* and biofilm-related gene *BCR1* (Fig. 4B).

### Global transcriptome of *Candida tropicalis* cells highlights the suppression of transcriptional machinery and rewiring of metabolism with upregulation of zinc metabolism genes in late response to miltefosine

#### Global transcriptional response of C. tropicalis to miltefosine

**Fig 5:**
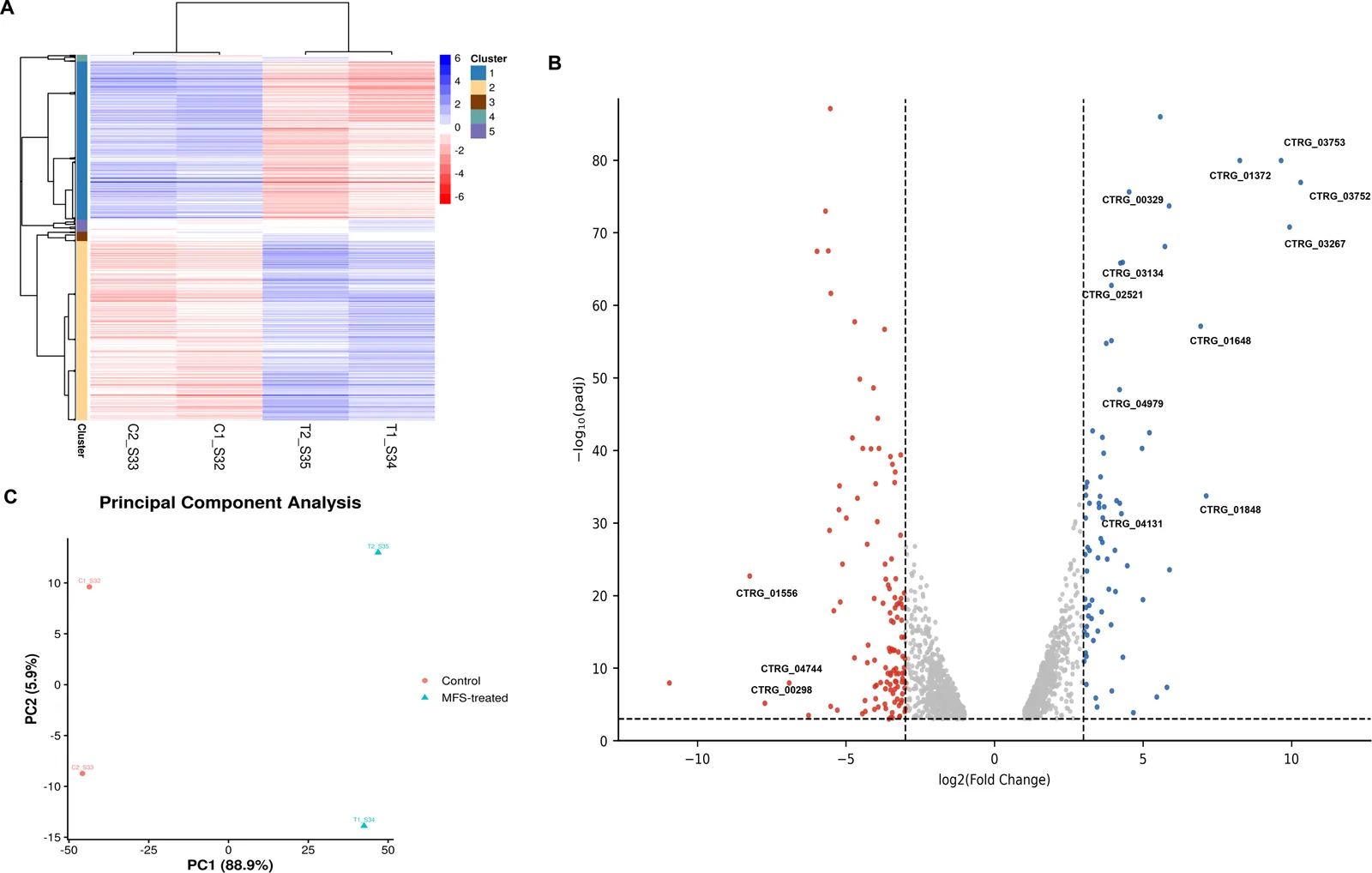
Heatmap, PCA, and volcano plot of DEGs in *Candida tropicalis* treated with miltefosine. (**A**) Heatmap showing the expression profile of DEGs, considering the biological replicates of untreated control (C1_S32 and C2_S33) and treated (T1_S34 and T2_S35) conditions, and rows to DEG genes. Expression values are scaled by gene (row z-score). Dendrograms represent hierarchical clustering of genes and samples. The colored side bars (clusters 1, 2, 3, 4, and 5) indicate five major gene clusters, grouping genes with similar expression patterns across the conditions used for subsequent functional enrichment analyses. (**B**) Volcano plot of differential expression (DESeq2) comparing MFS-treated versus untreated *C. tropicalis*. The x-axis shows log2(fold change) and the y-axis shows −log10(adjusted *P* value). Dashed vertical lines indicate the fold-change cutoff (|log2FC| ≥ 1.58) and the dashed horizontal line indicates the significance threshold (FDR ≤ 0.001). Blue points represent significantly upregulated genes in the MFS-treated condition, red points represent significantly downregulated genes, and gray points represent non-significant genes. (**C**) PCA was performed using normalized gene expression data obtained from RNA-seq clustered according to treatment condition, with control replicates clearly separated from MFS-treated replicates along PC1, indicating that MFS treatment was the primary source of transcriptional variation among the samples. Each point represents an independent biological replicate.

RNA-seq data showed 975 DEGs comparing untreated and MFS-treated cells, with 424 genes upregulated in the treatment condition and 551 genes upregulated in the untreated condition (Table S3). Hierarchical clustering of DEGs (|log2FC| ≥ 1.58, FDR ≤ 0.001) clearly separated untreated and MFS-treated *C. tropicalis* samples, indicating a robust and reproducible drug response (Fig. 5A). The MFS-treated replicates (T1_S34, T2_S35) were grouped together and away from the untreated replicates (C1_S32, C2_S33), while genes were segregated into five major expression clusters with opposite patterns between untreated and treated conditions.

The heatmap results suggest that MFS induced a coordinated repression of a large subset of biosynthetic and transport functions, while a smaller subset of stress- and signaling-related genes was induced (Fig. 5A). The largest groups (clusters 1 and 2) were strongly enriched in genes encoding membrane and cell-wall associated proteins, numerous transporters, and zinc-binding proteins, as well as components of RNA polymerase and other transcriptional regulators, whereas smaller clusters contain genes related to DNA repair, specific transcription factors, and enzymes involved in lipid and protein glycosylation.

The volcano plot (Fig. 5B) summarizes the magnitude and significance of transcriptional changes upon MFS exposure, showing a clear separation between significantly upregulated genes in treated cells (positive log2FC) and significantly downregulated genes (negative log2FC). Notably, several of the most strongly induced transcripts are linked to zinc homeostasis (including *PRA1* and zinc transporter-related loci), whereas repressed genes were broadly enriched in housekeeping and biosynthetic functions, consistent with the coordinated suppression of translational/transcriptional machinery highlighted by KEGG enrichment.

These patterns are consistent with what we observed in *C. albicans* and *C. tropicalis*: upregulation of filamentation repressors, such as CTRG_04979 (Log_2_FC: 2.07*—C. albicans* ortholog *NRG1*), CTRG_02521 (Log_2_FC: 2.54*—C. albicans* ortholog *WOR2*), and CTRG_03134 (Log_2_FC: 2.37*—C. albicans* ortholog: *CRZ2*), and strong induction of genes linked to zinc metabolism (e.g., *PRA1*) (Fig. 5B and Table S3), together with downregulation of filamentation-associated genes (*ECE1*, *HWP1*, *ALS* family, *EFG1*, *RAS1*, *CYR1*, *TEC1*, and *CEK1*) as observed in Fig. 4. In the current *C. tropicalis* data set, many DEGs fall into functional categories connected to these pathways (membrane transport, zinc binding, and cell-wall remodeling), supporting the idea that MFS acts both on morphogenesis circuits and on zinc homeostasis.

The PCA revealed that the major source of variation was associated with MFS treatment (Fig. 5C), as PC1 explained 88.9% of the total variance and clearly separated untreated and MFS-treated samples. Although some dispersion between biological replicates was observed along PC2, this component accounted for only 5.9% of the total variance and therefore represented a minor source of within-condition variability. Importantly, this secondary variation did not affect the clear separation of samples according to treatment condition.

#### KEGG enrichment points translation and core metabolism as major targets

**Fig 6:**
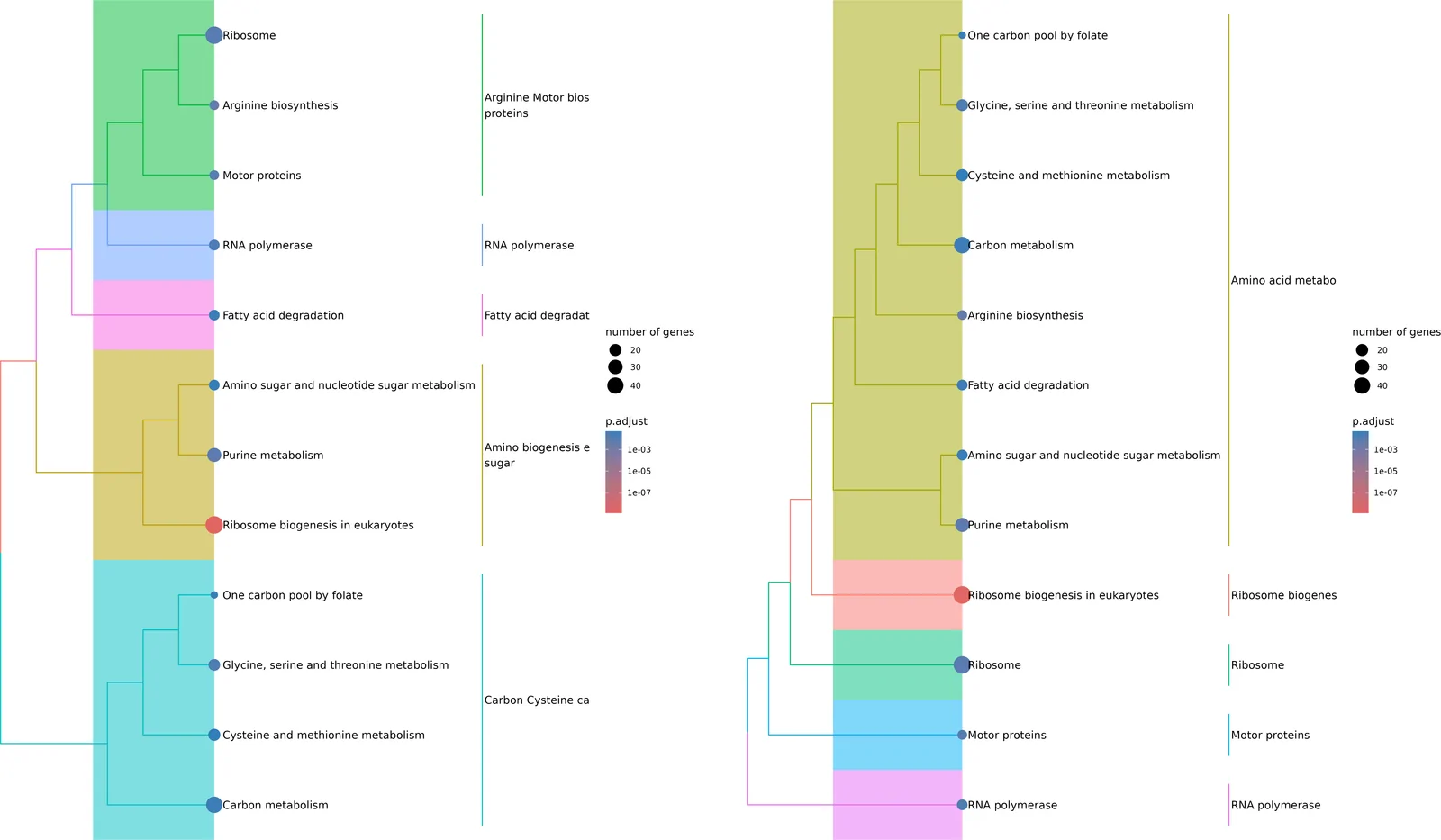
Functional grouping of KEGG-enriched pathways in miltefosine-treated *Candida tropicalis*. Treeplot showing hierarchical clustering of the most significantly enriched KEGG pathways based on gene overlap among DEGs in *C. tropicalis* treated with miltefosine *versus* untreated control. Each row corresponds to a KEGG pathway, and branches group pathways that share a larger proportion of differentially expressed genes.

KEGG analysis of the DEG set highlights a compact set of highly enriched pathways, based on a cutoff of *P* value ≤ 0.005 and FDR ≤ 0.05. The most significant categories are ribosome biogenesis in eukaryotes, ribosome, RNA polymerase, motor proteins, purine metabolism, carbon metabolism, glycine, serine, and threonine metabolism, arginine biosynthesis, fatty acid degradation, one-carbon pool by folate, amino sugar and nucleotide sugar metabolism, and cysteine and methionine metabolism (Fig. 6 and Fig. S6 through S8).

The Treeplot (Fig. 6) groups these KEGG terms into two main functional modules: (i) a “translation/transcription” module (ribosome, ribosome biogenesis, RNA polymerase, motor proteins), and (ii) a “core metabolism” module (carbon and amino acid metabolism, purine metabolism, one-carbon by folate, fatty acid degradation, amino sugar and nucleotide sugar metabolism, cysteine/methionine metabolism). These data are corroborated by Dotplot analysis (Fig. S6A), which indicated a coordinated downregulation of the translational machinery and associated transcriptional components in *C. tropicalis* exposed to MFS. This pattern is compatible with a classical stress/adaptation response in yeasts, where energy-expensive processes, such as ribosome production and bulk protein synthesis, are transcriptionally repressed during drug challenge (44, 45).

In contrast, several genes assigned to metabolic pathways, such as fatty-acid degradation, glycine/serine/threonine metabolism, one-carbon pool by folate, parts of carbon metabolism, and arginine biosynthesis, are enriched in blue, indicating upregulation of specific steps in these routes. This suggests a metabolic rewiring toward alternative carbon and nitrogen utilization and enhanced one-carbon/folate flux, which can support redox balance, nucleotide synthesis, and mitochondrial function under stress. Thus, the KEGG heatplot supports a model in which MFS simultaneously represses protein synthesis and reprograms core metabolism, rather than a non-specific global suppression (Fig. S6).

In addition, the UpSet plot (Fig. S7) confirmed that these pathways are highly interconnected: ribosome biogenesis and ribosome share the largest number of DEGs (~50 genes), and there is extensive gene overlap between purine metabolism, carbon metabolism, glycine/serine/threonine metabolism, fatty-acid degradation, and the folate one-carbon pool. This pattern is further reinforced by the Cnetplot (Fig. S8), where the same genes connect multiple pathways, showing suppression of translational machinery and rewiring of metabolism, such as ribosome biogenesis, purine metabolism, and carbon metabolism, forming a dense functional network.

#### Links to virulence, morphogenesis, stress response, and zinc/metal metabolism

Although KEGG enrichment is dominated by metabolic and translational terms, the DEG list and cluster annotation indicate that MFS also impacts multiple virulence-related processes in *C. tropicalis*, in agreement with previous reports showing that MFS interferes with biofilm formation, cell surface properties, and stress pathways in *Candida* (18, 19, 46, 47). Clusters enriched in DEGs show a high density of genes annotated to cell wall, plasma membrane, transmembrane transport, protein glycosylation, and amino-sugar metabolism, all central for cell wall biosynthesis and remodeling. This is consistent with previous evidence that MFS perturbs membrane lipids and cell wall integrity in *Candida* (19, 46, 48).

Several DEGs are associated with oxidative and osmotic stress responses and mitochondrial function, in line with reports that MFS increases ROS, alters mitochondrial membrane potential, and can trigger apoptosis-like death in fungi (47). The enrichment of fatty acid degradation and one-carbon/folate-related pathways fits a scenario where cells adjust central metabolism to sustain redox balance and energy production under drug stress. The DEGs annotated with roles in biofilm formation and adhesion (including genes involved in vesicle-mediated transport and surface remodeling) link the transcriptomic response to the antibiofilm and anti-adhesion phenotypes observed for MFS. Even though “morphogenesis” is not a KEGG term, these data integrate well with your earlier findings that MFS downregulates hyphae-associated genes and upregulates transcriptional repressors of filamentation (e.g., *NRG1*, *WOR2*, *CRZ2*, Table S2), supporting the inhibition of yeast-to-hyphae transition as a conserved component of the response (49).

**Fig 7:**
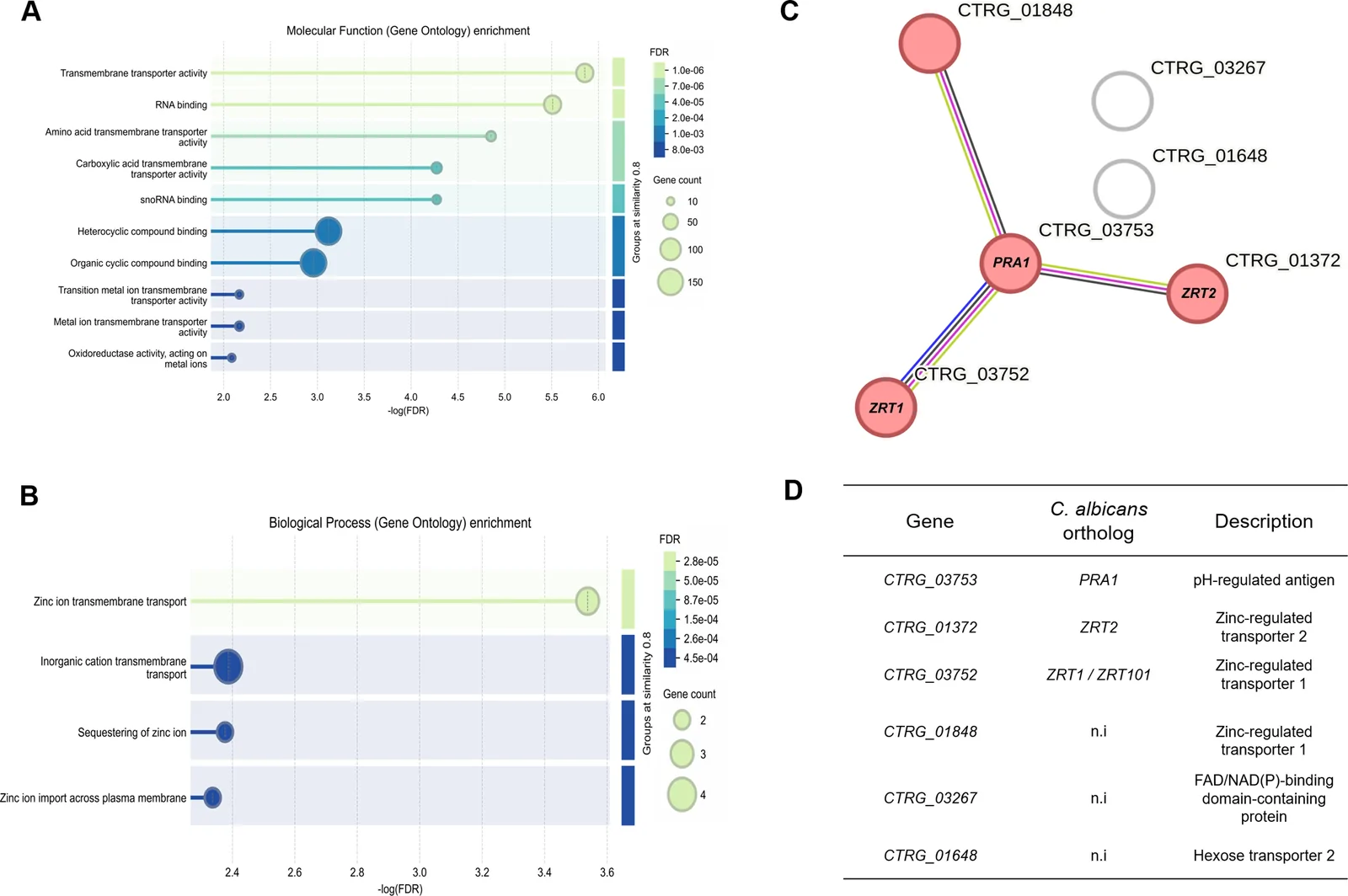
Comparisons of transcriptional profiles across MFS treated and untreated *Candida tropicalis* reveal zinc metabolism enrichment machinery. (**A**) Gene Ontology Enrichment analysis based on STRINGdb, with a cutoff of |log_2_(FC)| > 3 and *P*-adjusted value ≤ 0.001 for global action based on molecular function categories and (**B**) biological processes categories based on STRINGdb, with a stricter cutoff of |log2(FC)| > 7 and *P* value ≤ 0.001. (**C**) The sub-network represents a selection of high-confidence interactions (score between 0.414 and 0.952) from the full STRINGdb network on upregulated DEGs categorized by their biological processes. The nodes represent all the proteins produced by a single, protein-coding gene locus. Connection lines represent co-occurrence (blue), co-expression (black), experimental data (purple), and co-mentioned in PubMed abstracts (yellow). StringDB and Uniprot were used to generate GO maps and gene names. Panel **D** represents the genes highlighted as orthologs with *C. albicans* from *Candida* genome database. n.i., not identified.

Gene Ontology analysis showed major molecular function recruitment to MFS response for transmembrane transporter activity and RNA binding (Fig. 7A). Focusing on more specific traits in this phenomenon, the Fig. 7B data emphasizes zinc metabolism, mainly zinc transport across the cell membrane. Six genes were underlined when we established a more stringent cutoff to identify which genes are most upregulated in *C. tropicalis* cells during MFS treatment (i.e., log_2_FC > 7 and *P* value ≤ 0.001): CTRG_03753 (*PRA1* ortholog in *C. albicans*), CTRG_01372 (*ZRT2* ortholog in *C. albicans*), CTRG_03752 (*ZRT101/ZRT1* ortholog in *C. albicans*), and CTRG_03267, CTRG_01648, CTRG_01848, for which no orthologous genes have been identified in *C. albicans* according to *Candida* Genome Database (Fig. 7C and D).

Therefore, the clusters are enriched in zinc-binding proteins and genes involved in metal transport and homeostasis, consistent with earlier observation of *PRA1*/zincophore induction. Together, these results reinforce the idea that disturbance of zinc/metal homeostasis may be associated with changes in cell surface, biofilm behavior, and stress adaptation in MFS-treated *Candida* cells (50, 51). Finally, the enrichment of DEGs annotated to cell wall and plasma membrane, amino-sugar and nucleotide-sugar metabolism, transporters, and zinc-binding proteins can indicate that MFS also remodels the cell surface and metal homeostasis networks. The zinc acquisition system in *C. albicans* is known to influence biofilm formation and virulence, directly linking zinc homeostasis to cell-surface behavior (51). In parallel, MFS has been shown to inhibit *Candida* biofilm formation, further supporting a connection between its action on membranes/metal homeostasis and attenuation of biofilm-associated virulence traits.

### Zinc may be important to the adaptation of *Candida tropicalis* under miltefosine exposure through upregulation of zincophores and zinc transporters

**Fig 8:**
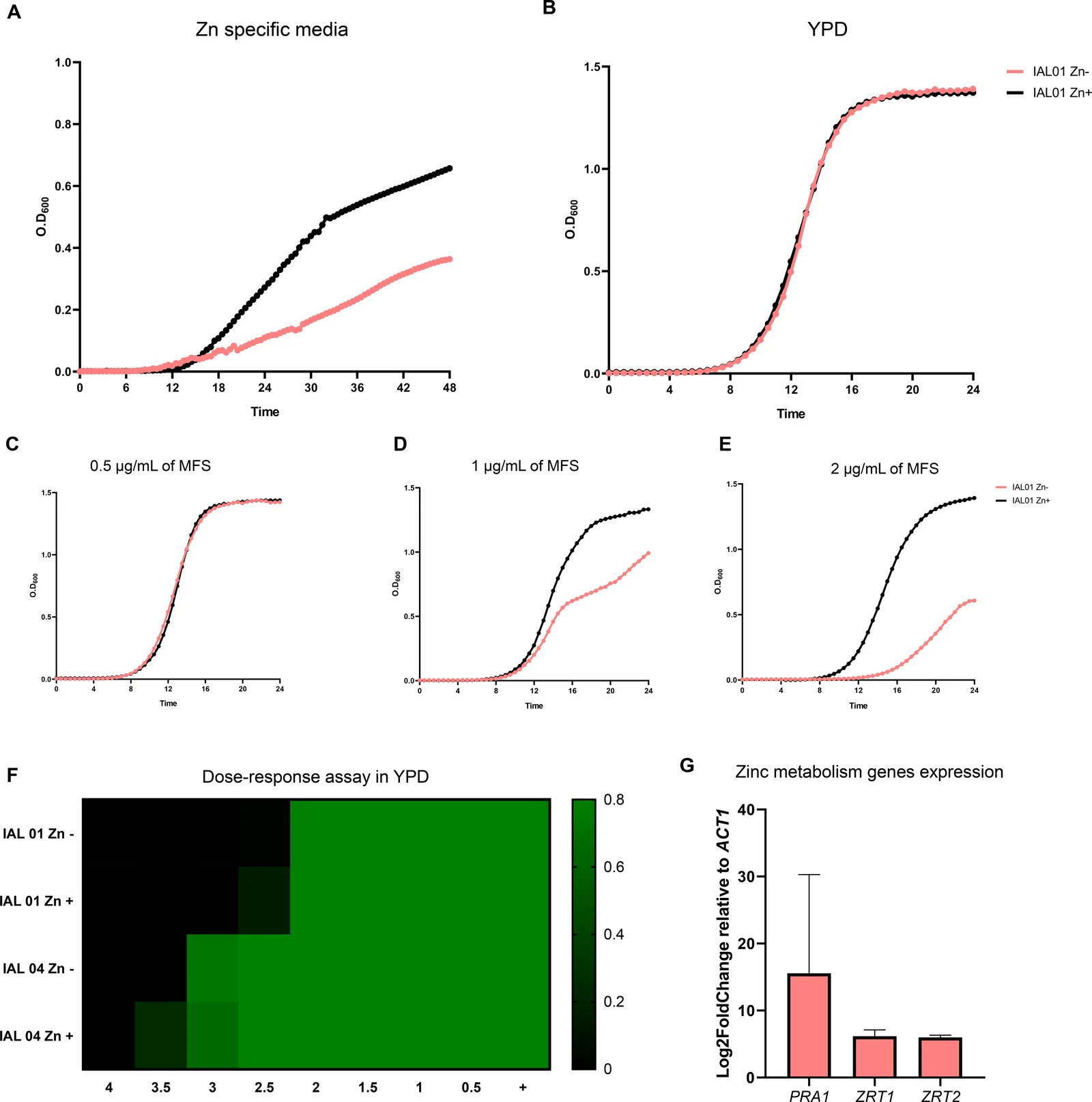
Impact of zinc availability on the growth and miltefosine tolerance in *Candida tropicalis* IAL-01 isolate. (**A**) Growth curve in zinc-specific medium with or without ZnSO₄·7H₂O at 9 µM. (**B**) Growth curve in YPD broth with or without ZnSO₄·7H₂O at 9 µM. (**C–E**) Growth curves in YPD with or without ZnSO₄·7H₂O supplemented with 0.5, 1, and 2 μg/mL MFS, respectively. (**F**) Dose-response assay of *C. tropicalis* exposed to increasing concentrations of MFS (0.5–4 μg/mL in YPD with or without ZnSO₄·7H₂O at 9 µM). (**G**) Relative expression of zinc metabolism-related genes *PRA1*, *ZRT1*, and *ZRT2* under MFS treatment.

To further validate the transcriptomic data, particularly of an orchestrated zinc-related metabolism shift under MFS treatment, growth curves of *C. tropicalis* in zinc-supplemented media were obtained to analyze if zinc availability can improve *C. tropicalis* growth upon exposure to MFS. Indeed, Fig. 8A showed that *C. tropicalis* grew better in defined media with zinc supplementation (zinc-specific media). Otherwise, using YPD broth as a rich medium does not change the growth capacity of the isolate with or without zinc supplementation (Fig. 8B). As the *C. tropicalis* growth in zinc-specific media is slower, the YPD broth was used to evaluate the impact of zinc availability on the response to MFS. Strikingly, under MFS subinhibitory concentrations, zinc supplementation in media significantly enhanced fungal growth (Fig. 8C through E). These data corroborate the mean optical density shown in Fig. 8F, where fungal growth was observed at 2.5 and 3.5 µg/mL of MFS when zinc is available in the medium, but not in the absence of zinc for both *C. tropicalis* isolates. Finally, to validate RNA-seq data of the zinc-metabolism shift, the genes for zincophore *PRA1* and zinc transporters *ZRT1* and *ZRT2* were upregulated under MFS treatment (Fig. 8G).

## DISCUSSION

Our results showed the *in vitro* inhibitory and fungicidal effects of MFS on *C. albicans* and *C. tropicalis* isolates and antibiofilm activity across distinct development phases (adherence, biofilm formation, and pre-formed biofilms), corroborating previous data (16). The inhibition of yeast-to-hyphae transition by MFS was also observed here, and this effect has been consistently described in previously published *in vitro* and *in vivo* studies supporting the MFS repurposing as a candidate antifungal for candidiasis treatment (17, 18, 20–22). In fact, inhibition of the yeast-to-hyphae transition is a relevant antifungal target, since the filamentous form plays a key role in enhancing adhesion, biofilm formation, tissue invasion, and immune system evasion (52). To advance our understanding, integrating a global transcriptomic analysis with virulence gene expression data was essential to reveal the molecular basis of MFS activity in inhibiting filamentation and biofilm formation in hyphae-forming *Candida* species, especially *C. tropicalis*.

It was observed that MFS at subinhibitory concentrations in filamentation-inducing media (such as RPMI, Spider, Lee, and SM-GlcNAc) inhibited the morphogenesis of *C. albicans* and *C. tropicalis*. In addition, these results are corroborated by the relative gene expression of virulence factors in *C. albicans* and *C. tropicalis* treated with MFS. The drug induced downregulation of several genes, such as those involved in adhesion, biofilm formation, and morphogenesis, but we highlighted the downregulation of the genes associated with main filamentation-inducing pathways: *EFG1*, *TEC1*, *NGT1*, *RAS1*, *CYR1*, *CEK1*, and *NGT1* for *C. albicans* and *BCR1*, *EFG1*, and *NGT1* for *C. tropicalis*.

The results suggested that MFS can reduce gene expression of components involved in the morphogenesis signaling cascade (*RAS1*, *CYR1*, *EFG1*, and *TEC1*) (32, 35). In addition, *EFG1*, *TEC1*, *CPH1*, and *CPH2* also participate in the hyphal elongation process (53), which was negatively regulated by the action of MFS in *C. albicans* (*EFG1*, *TEC1*, *CPH1*, and *CPH2*) and *C. tropicalis* (*EFG1*).

The mitogen-activated protein kinase (MAPK) pathways are comprised of four pathways identified in *C. albicans*: the high osmolarity glycerol (HOG) pathway, the Mkc1 cell wall integrity pathway, and the Cek1 and Cek2 pathways (*Candida* ERK-like kinase), involved in adaptation to osmotic and oxidative stresses, repair and biogenesis of the cell wall, biofilm formation, hyphal development, and virulence (54–57). The *CEK1* gene was downregulated by MFS, which may be associated with cell wall remodeling and impairing hyphal development.

Here, we also found the upregulation of three filamentation repressors, *NRG1*, *WOR2*, and *CRZ2*, *C. albicans* orthologs (58, 59) in RNA-seq analysis, possibly acting in *C. tropicalis* as an additional mechanism of filamentation blocking. Taken together, our results suggest that MFS inhibits the yeast-to-hyphae/pseudohyphae transition and that the genetic network related to cAMP- and MAPK signaling pathways may be involved.

The downregulation of some genes associated with other virulence factors of *C. albicans* and *C. tropicalis* was also observed after MFS exposure. The peptide toxin candidalysin, encoded by the *ECE1* gene, appears to be intrinsically related to hyphae formation, and the downregulation seen in *C. albicans* may be caused by the downregulation of genes involved in the filamentation signaling cascades (60). During biofilm formation, Bcr1 acts as a key regulator of adhesin expression, such as Hwp1 protein and agglutinin-like sequence (Als) family proteins in the filamentous form (61). In our study, we observed the downregulation of *HWP1* and *ALS3* in *C. albicans* and *ALS1* in *C. tropicalis*. Then, MFS-mediated response led the cell to a loss in the process of adhesion and biofilm formation in *C. albicans* (62), as we observed in our data that MFS inhibited adhesion on polystyrene and biofilm formation of *C. albicans* and *C. tropicalis*, consistent with our phenotypic data showing reduced adhesion on polystyrene and impaired biofilm formation in both species.

Regarding the fungal cell wall, MFS affected the morphology and structural components during inhibition of the yeast-to-hyphae transition in *C. albicans* and *C. tropicalis*. Vila et al. (18) described a thickening of the *C. albicans* cell wall when treated with MFS at sub-MIC, while Wu et al. (47) showed a thinner cell wall under treatment with fungicidal concentration by transmission electron microscopy. Moreover, MFS also altered the exposure of the structural components (chitin/chitosan) of *C. albicans* and *C. tropicalis* cell walls. For *C. albicans*, around 5% of the chitin content is deacetylated to chitosan by the chitin deacetylase enzyme, making the chitin fibrils more elastic, protecting them from the action of chitinases, and contributing to stress resistance (63–65). It is also known that the response to cellular damage and chitin synthesis comprises the Protein kinase C, HOG, and calcium-calcineurin signaling pathways (66, 67). In stress conditions, the general response of the cell wall is increased chitin (68), as described in *Candida* isolates tolerant to echinocandin (69), and observed here for both *Candida* species treated with MFS.

Moreover, several works have demonstrated that MFS induces loss of plasma membrane integrity and permeability in fungi (16), by direct interaction with ergosterol as described in *Cryptococcus* spp. (70), and/or by total ergosterol reduction in *Coccidioides posadasii* and *Histoplasma capsulatum* (71). Here, we did not observe significant changes in *ERG11* expression, which suggests that MFS may not have interfered with ergosterol biosynthesis in *C. albicans* and *C. tropicalis*, as Wu et al. (72) showed in ergosterol quantification and *ERG11* expression in *Nakaseomyces glabratus* (formerly *Candida glabrata*) treated with MFS. In contrast, the authors described that zymosterol accumulation caused by the deletion of the *OSH2* gene can mediate MFS resistance (72). Another interesting key point noted in our data were the enrichment of the sphingolipid biosynthesis genes CTRG_04131 (Log_2_FC: 2.92*—C. albicans* ortholog orf19.4779) and CTRG_00329 (Log_2_FC: 3.56*—C. albicans* ortholog orf19.1308) (Table S3), which can suggest that MFS may affect sphingolipid homeostasis of the cell membrane, as well as observed previously in *Aspergillus fumigatus* (73) and in protozoans, where MFS disturbs membrane homeostasis by decreasing many membrane phospholipids and increasing sphingolipids and sterols content (74), suggesting an evolutionary conservation of the MFS action among eukaryotes.

Understanding MFS action with a global analysis, Wu et al. (47) applied transcriptomic and proteomic approaches, highlighting Aif1 (an apoptosis-inducing factor) as the main response to MFS in *C. albicans*, inducing cell death, and it was strongly correlated with increased oxidative stress and decreased mitochondrial membrane potential. We can consider this Aif1 action as a major component in an early response to MFS, due to their experiments conducted with *C. albicans* cells treated with a fungicidal concentration of MFS at 30°C for 2 h, a different approach from ours. Here, we treated *C. tropicalis* yeasts with a subinhibitory concentration of MFS at 35°C for 20 h to get only the yeast form. Therefore, both approaches are complementary to understand the molecular mechanisms for *Candida* spp. under the inhibitory and fungicidal actions of MFS.

After global analysis of transcriptome data, we may highlight a broad modulation of genes involved in carbohydrates, amino acid, and lipid metabolic pathways, but especially genes involved in the zinc metabolism for MFS late response, with *PRA1* (pH-regulated antigen 1, a cell surface protein that sequesters zinc from host tissue) and zinc transporters (*ZRT1* and *ZRT2*) as the principal upregulated genes. This zinc supplementation in media enhanced fungal growth under MFS treatment when compared with growth in zinc-limited media. These data, combined with RNA-seq results validated by the relative gene expression of *PRA1*, *ZRT1*, and *ZRT2*, may reveal the zinc metabolism as an adaptive mechanism of *C. tropicalis* to endure the presence of MFS. Moreover, the importance of zinc assimilation is described as crucial for *C. albicans* pathogenicity, and in the same way, zinc limitation induces Pra1 zincophore protein to scavenge zinc from the host and interacts with the Zrt1 transporter during host-pathogen interaction (75, 76).

Still in *C. albicans*, the main classes of transcription factors are the zinc cluster proteins, defined as a cysteine-rich region that drives zinc as part of the DNA-binding domain of the protein and is also conserved in eukaryotes as essential regulators of gene expression (77, 78). An important transcription factor, Csr1 (also Zap1 in *C. albicans*), directly regulates the expression of genes responsible for zinc uptake, such as the transporters Zrt1 and Zrt2, and the zinc-binding protein Pra1 (79, 80). Interestingly, Ramírez-Zavala et al. (81) demonstrated that activated forms of both Tac1 and Znc1 (zinc cluster transcription factors) are Rta3-dependent, resulting in increased MFS resistance. This finding was corroborated when Rta3, a seven-transmembrane receptor protein involved in the regulation of asymmetric lipid distribution in the plasma membrane, was deleted in the strain, leading to hypersensitivity to MFS (81).

The wide distribution of the zinc-controlled network leads us to explore potential links between filamentation block and zinc metabolism, mainly Pra1 protein, although the role of zinc in *C. albicans* morphology is not well understood (82). Malavia et al. (83) found that a lack of *PRA1* leads to blocked cell enlargement and adhesion (83). In addition, Pra1 functions as a ligand for the αMβ2 integrin and promotes neutrophil recruitment during systemic *C. albicans* infection (83). In a mouse model of systemic candidiasis, the *pra1Δ* mutant exhibited hypervirulence due to impaired neutrophil recruitment. Since neutrophils exert strong antifungal activity, the absence of the *PRA1* gene or its reduced expression in many human fungal pathogens may represent an effective strategy for immune evasion (84). Previously, Yamaguchi (28) had reported that zinc depletion may induce *C. albicans* filamentation, and Kim et al. (79) described that a mutant strain for *CRS1* under zinc-limited growth conditions presented a filamentation defect. Based on the literature and our data, it is possible to suggest that the superexpression of zinc transporters and zincophore may be an additional response of *Candida* cells during MFS late exposure, beyond the oxireduction disturbance and apoptosis induction previously described.

In a sense, the mechanism of action of MFS is not fully understood, but studies have highlighted that it may act on multiple targets (16). In this regard, although our study has provided new molecular insights into the mechanisms of action of MFS, we recognize the existence of some limitations: biochemical quantification of the cell wall components to confirm the imbalance observed using fluorescent markers, an increase in biological replicates in the RNA-seq experiment to improve the statistical power, and testing more than one isolate from each filament-forming *Candida* species to evaluate the transcriptome profile under MFS-induced stress conditions. In addition, this study fosters reflection on the impact of zinc metabolism on the fungal cell response to MFS-induced stress, opening perspectives to perform a proteomic analysis to correlate the transcriptomic and phenotypic profiles observed in MFS-treated *C. tropicalis* and, at long last, to include deletion mutants for, especially, *PRA1*, *ZRT1*, and *ZRT2* genes.

In conclusion, MFS inhibits fungal growth, yeast-to-hyphae transition, and biofilm formation in association with downregulation of gene expression related to virulence factors and biofilm formation. Additionally, the effects of MFS extend beyond the previously reported mitochondrial damage and ROS-induced apoptosis, encompassing significant upregulation of zinc acquisition machinery, especially zinc transporters and Pra1, an important zincophore related to *Candida* pathogenesis. Moreover, our findings help bridge the knowledge gap on how MFS modulates morphogenesis in true hyphae-producing *Candida* species.

## Data Availability

The RNA sequences were deposited in the NCBI database under number PRJNA1400329. In addition, all data will be made available from the corresponding author upon request.

## SUPPLEMENTAL MATERIAL

Supplemental Materials spectrum.00519-26-s0001.pdf Table S2; Fig. S1 to S8, and Supplemental References.

Table S1 spectrum.00519-26-s0002.xlsx List of primers and description of *Candida albicans* genes evaluated in the study.

Table S3 spectrum.00519-26-s0003.xlsx Candida tropicalis differentially expressed genes (DEGs) upon exposure to a subinhibitory concentration of miltefosine.

ASM does not own the copyrights to Supplemental Material that may be linked to, or accessed through, an article. The authors have granted ASM a non-exclusive, world-wide license to publish the Supplemental Material files. Please contact the corresponding author directly for reuse.
