# Supplementary material for: Miltefosine regulates gene expression related to morphogenesis and zinc metabolism in Candida albicans and Candida tropicalis
Source: Microbiol Spectr. 2026 Aug 26;14(10):e00519-26. doi: 10.1128/spectrum.00519-26 (PMC13689233; doi:10.1128/spectrum.00519-26)
Supplement: Supplementary file 1 [file spectrum.00519-26-s0001.pdf]

Supplementary material

Miltefosine regulates gene expression related to morphogenesis and zinc metabolism in *Candida albicans* and *Candida tropicalis*

Murilo Moreira dos Santos[a], Vinícius de Morais Barroso[a], Sofia Prampero[b], Márcia Cristina Furlaneto[c], Milton Yutaka Nishiyama-Jr[b], Kelly Ishida[a#]

[a]Laboratory of Antifungal Chemotherapy, Department of Microbiology, Institute of Biomedical Sciences, University of São Paulo, São Paulo, Brazil.

[b]Laboratory of Applied Toxinology, Butantan Institute, São Paulo, Brazil.

[c]Department of Microbiology, Center of Biological Sciences, State University of Londrina, Paraná, Brazil.

Running Head: Miltefosine controls morphogenesis and zinc in *Candida*

#Address correspondence to Kelly Ishida, ishidakelly@usp.br

Table S2. Minimum inhibitory concentration (MIC) and minimum fungicidal concentration (MFC) of miltefosine (MFS) in four different filament-inducing media on *Candida albicans* (SC5314) and *Candida tropicalis* IAL-01).

**Strains**	**Miltefosine MIC/MFC (μg/mL)**			
	**RPMI-MOPS**	**Lee**	**Spider**	**SM-GlcNAc**
SC5314	2/2	1/1	2/2	1/2
IAL-01	4/8	4/4	4/4	4/4

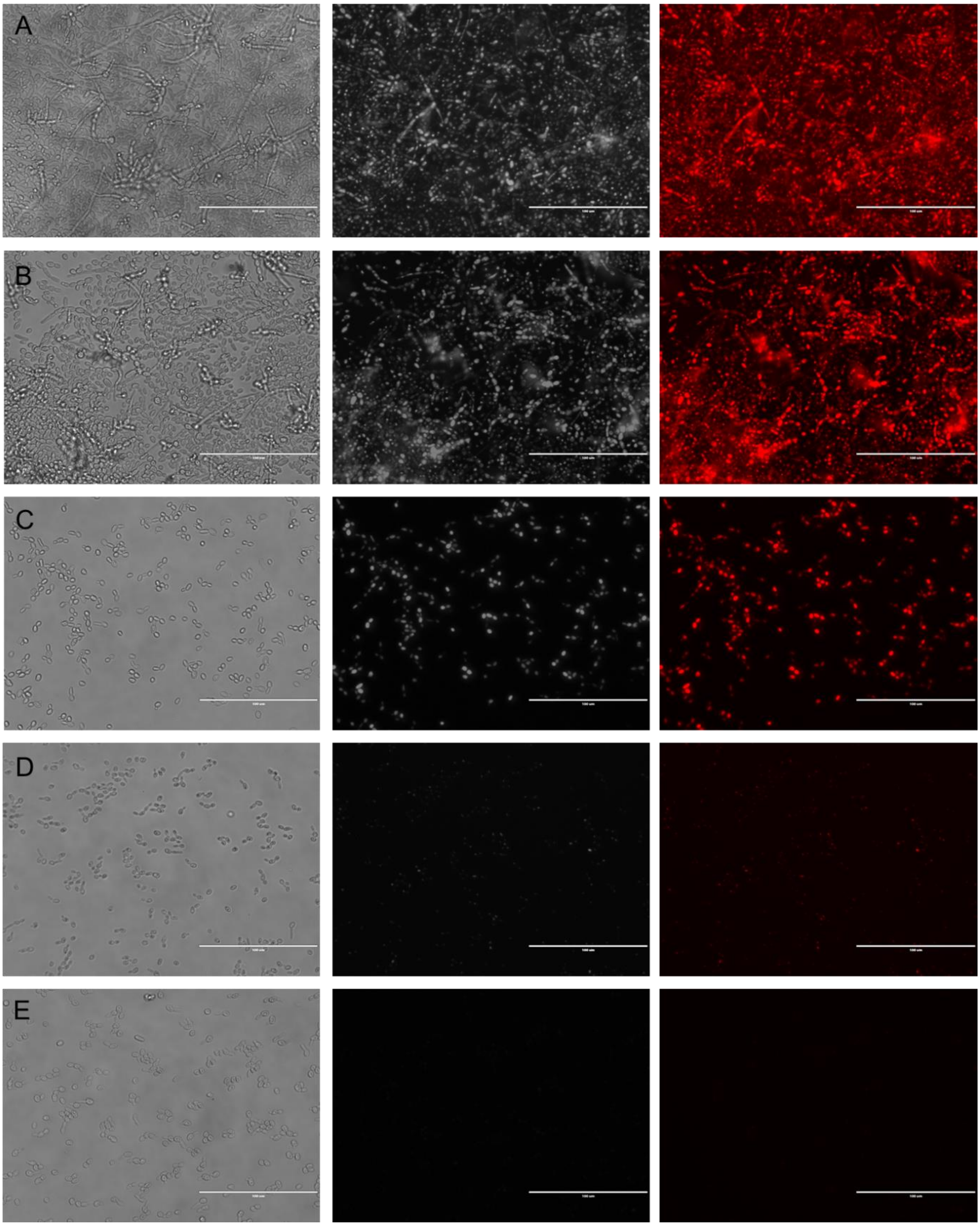

Figure S1. Miltefosine can eradicate ***Candida albicans*** **biofilm formation.** Light and fluorescence microscopy images of biofilms, in formation, of *Candida albicans* SC5314 untreated and treated with miltefosine. The images on the left are from light microscopy, the central images are marked with Calcofluor White, and on the right marked with FUN-1. (A) untreated; (B) 1 µg/mL; (C) 2 µg/mL; (D) 4 µg/mL and (E) 16 µg/mL. Bars = 100 µm.

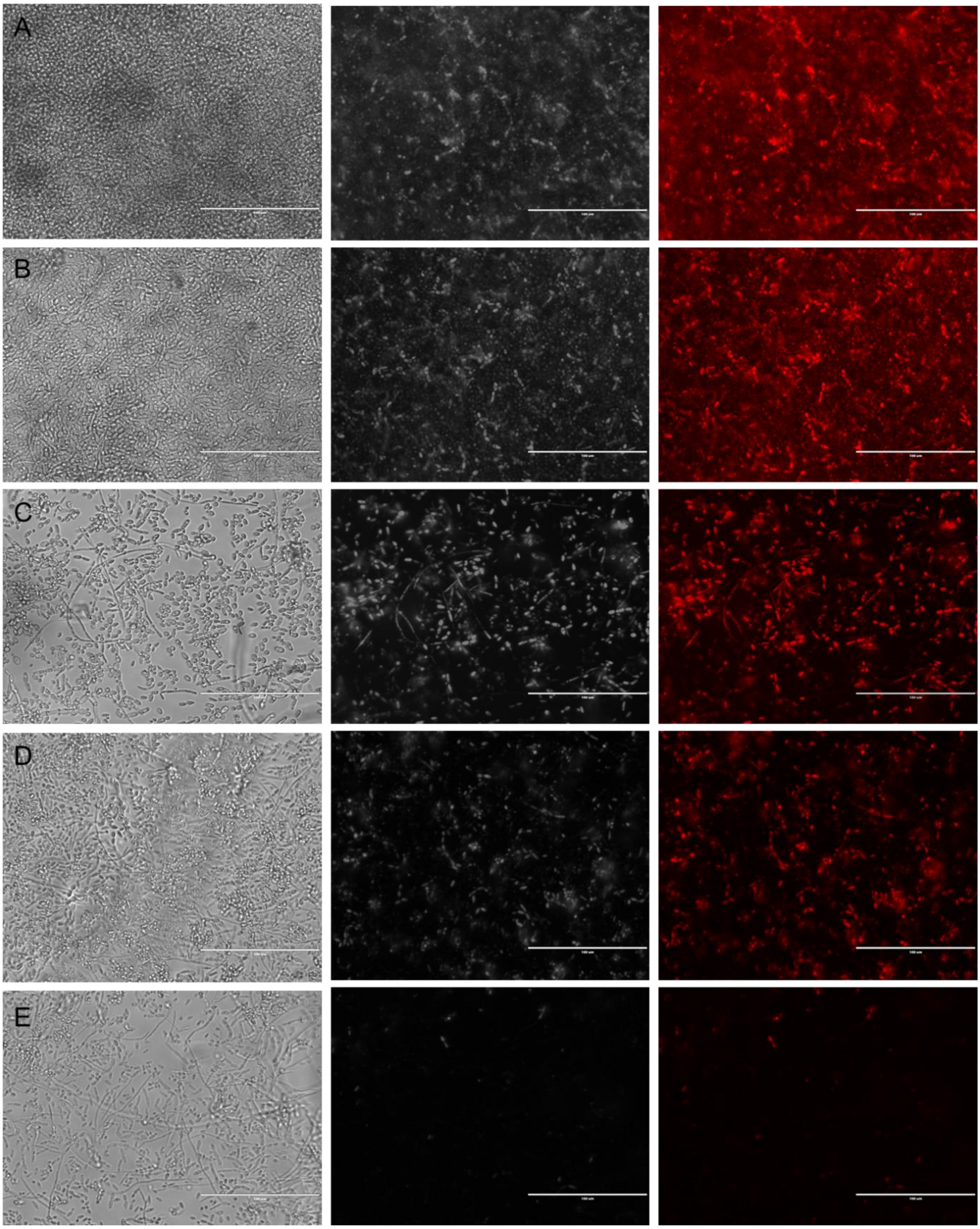

Figure S2. Miltefosine can eradicate *Candida albicans* mature biofilms. Light and fluorescence microscopy images of mature biofilms of *Candida albicans* SC5314 untreated and treated with miltefosine. The images on the left are from light microscopy, the central images are marked with Calcofluor White and on the right marked with FUN-1. (A) untreated; (B) 1 μg/mL; (C) 16 μg/mL; (D) 64 μg/mL; (E) 128 μg/mL. Bars = 100 μm.

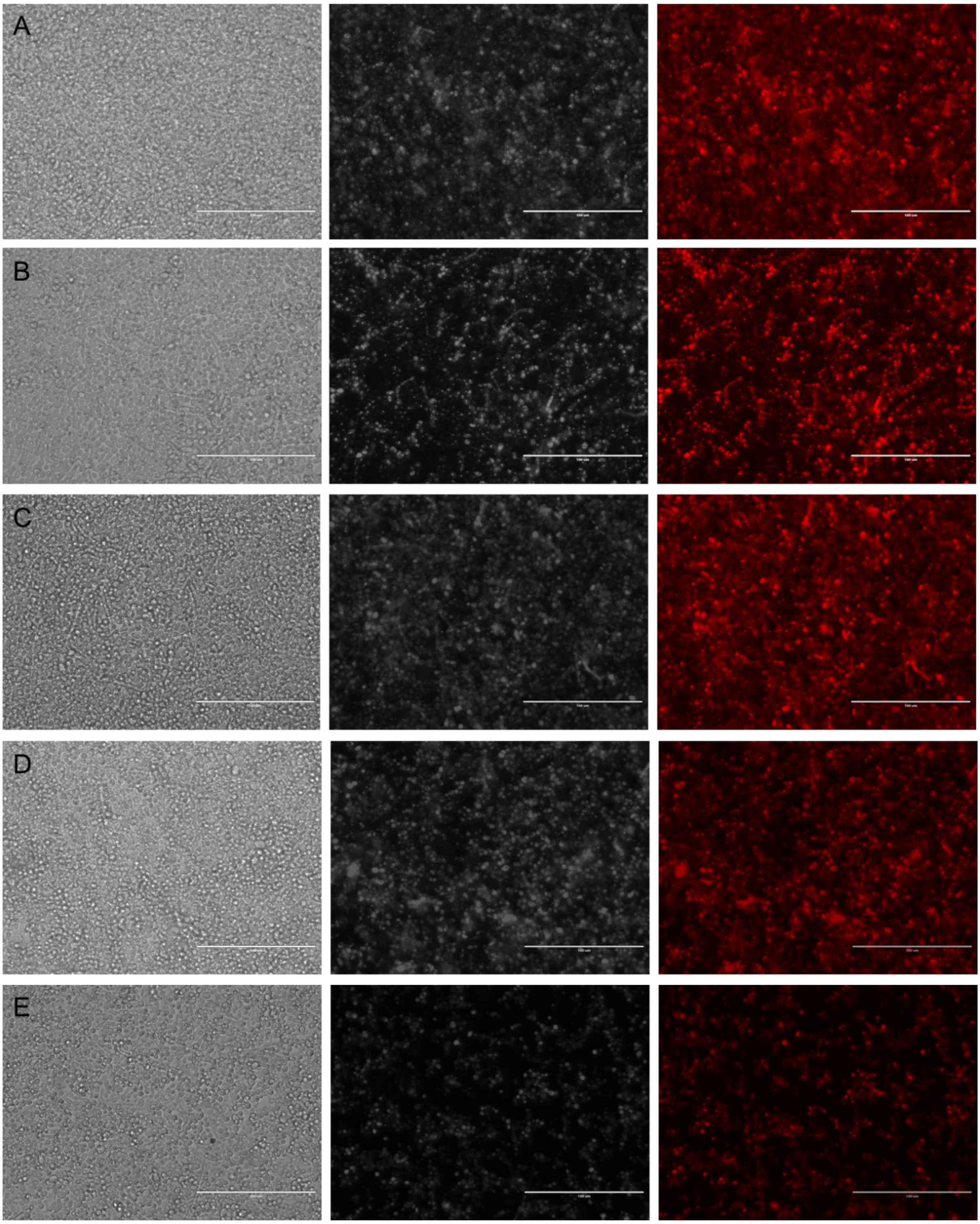

Figure S3. Miltefosine can eradicate *Candida tropicalis* biofilm formation. Light and fluorescence microscopy images of biofilms, in formation, of *Candida tropicalis* IAL-01 untreated and treated with miltefosine. The images on the left are from light microscopy, the central images are marked with Calcofluor White and on the right marked with FUN-1. (A) untreated; (B) 1 μg/mL; (C) 2 μg/mL; (D) 4 μg/mL and (E) 16 μg/mL. Bars = 100 μm.

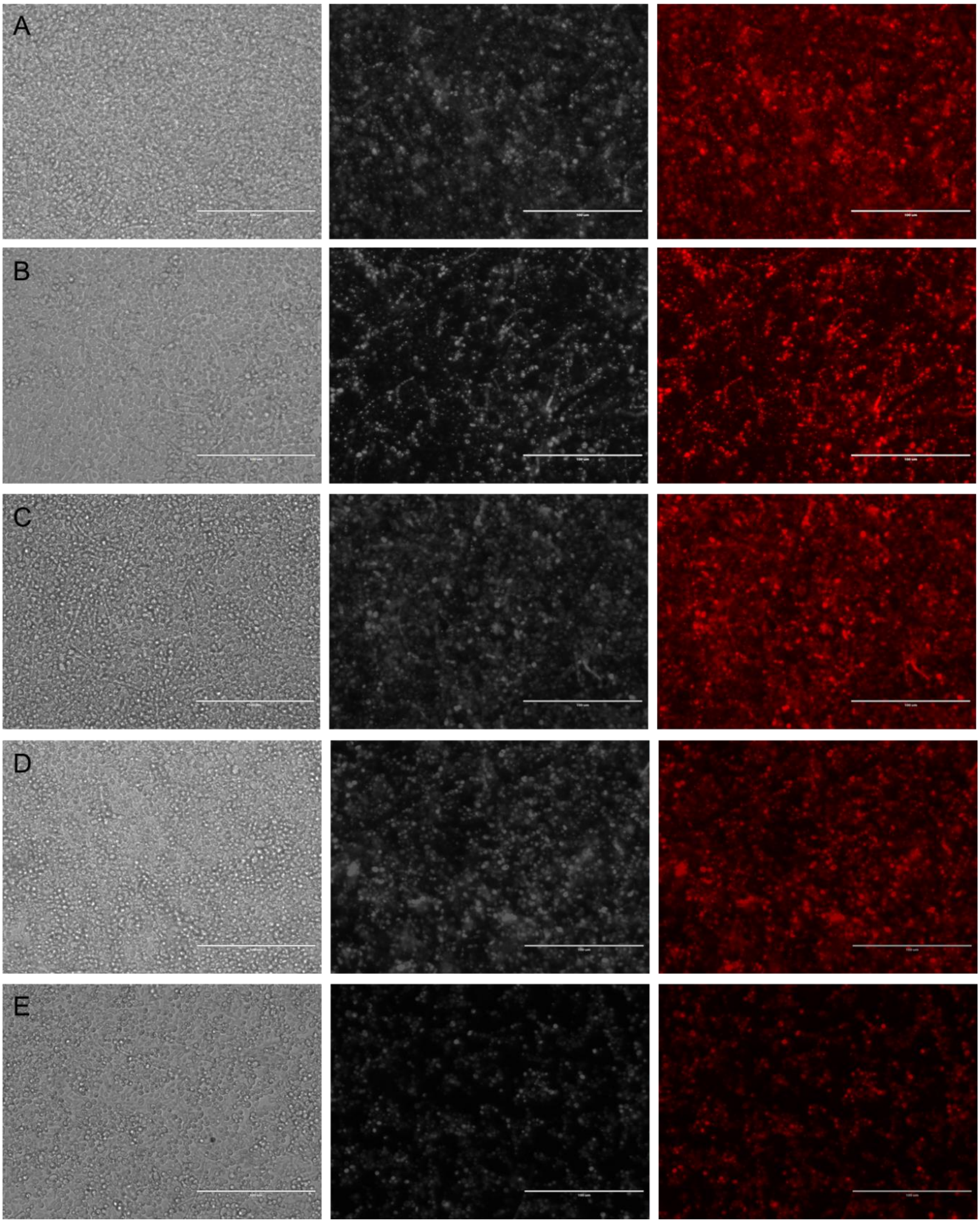

Figure S4. Miltefosine can eradicate *Candida tropicalis* mature biofilms. Light and fluorescence microscopy images of mature biofilms of *Candida tropicalis* IAL-01 untreated and treated with miltefosine. The images on the left are from optical microscopy, the central images are marked with Calcofluor White and on the right marked with FUN-1. (A) untreated. (B) 1 µg/mL; (C) 16 µg/mL; (D) 64 µg/mL; (E) 128 µg/mL. Bars = 100µm.

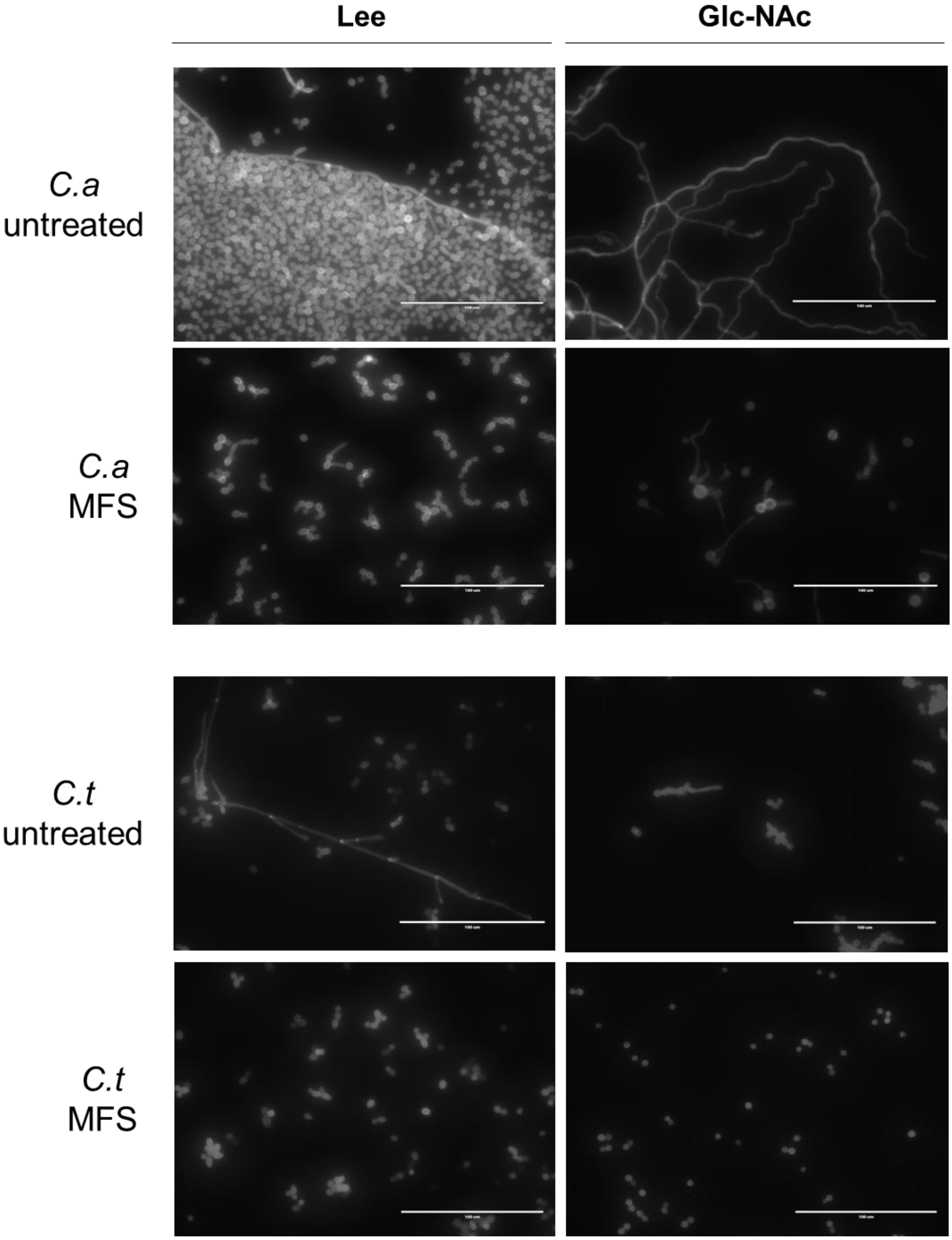

Fig. S5. Miltefosine inhibits filamentation in different filament-inducing media. Fluorescence microscopy of *Candida albicans* SC5314 (*C.a*) and *Candida tropicalis* IAL-01 (*C.t*) treated or not with a subinhibitory concentration of miltefosine (MFS) in Lee's glucose media and SM-supplemented with N-acetylglucosamine. Each row represents *C. albicans* in the untreated condition, *C. albicans* treated with MFS, *C. tropicalis* in the untreated condition and *C. tropicalis* treated with MFS, respectively. Bars = 100 µm.

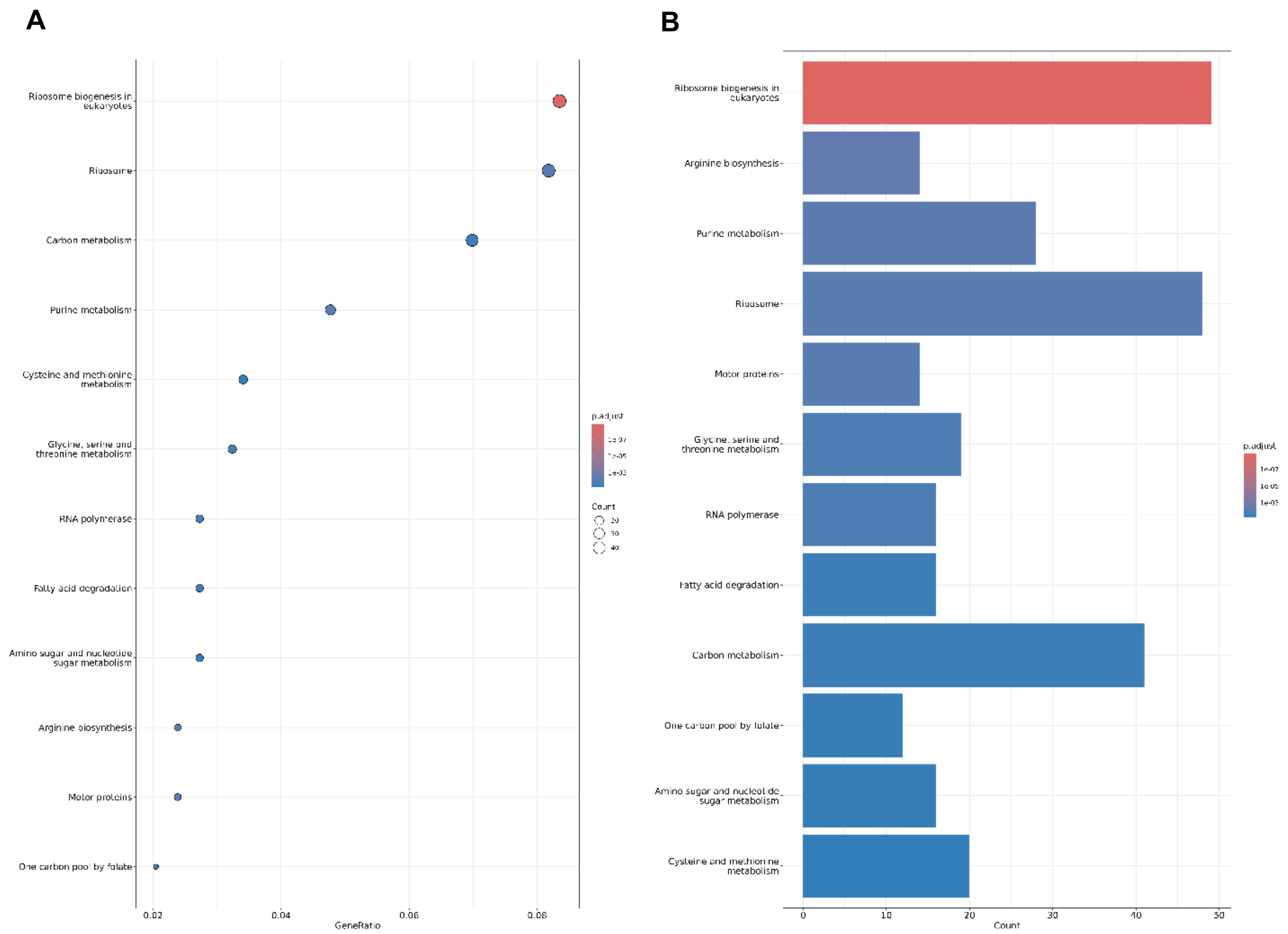

Figure S6. KEGG dot plot and heatplot of DEGs in enriched pathways in miltefosine-treated *Candida tropicalis*. (A) Dot plot showing the top KEGG pathways enriched in *C. tropicalis* treated with miltefosine compared with the untreated control in RPMI. Each dot represents a pathway; the x-axis indicates the GeneRatio (number of DEGs in the pathway divided by the total number of DEGs in the analysis). Dot size corresponds to the number of DEGs mapped to each pathway (Count), and dot color encodes the adjusted *p*-value (p.adjust), with red indicating more significant enrichment and blue less significant enrichment. (B) Heatplot showing the $\log_2$ fold change of DEGs mapped to the main KEGG pathways enriched in *C. tropicalis* treated with miltefosine *versus* untreated control in RPMI. Each row corresponds to a KEGG pathway, and each vertical bar represents a single DEG assigned to that pathway.

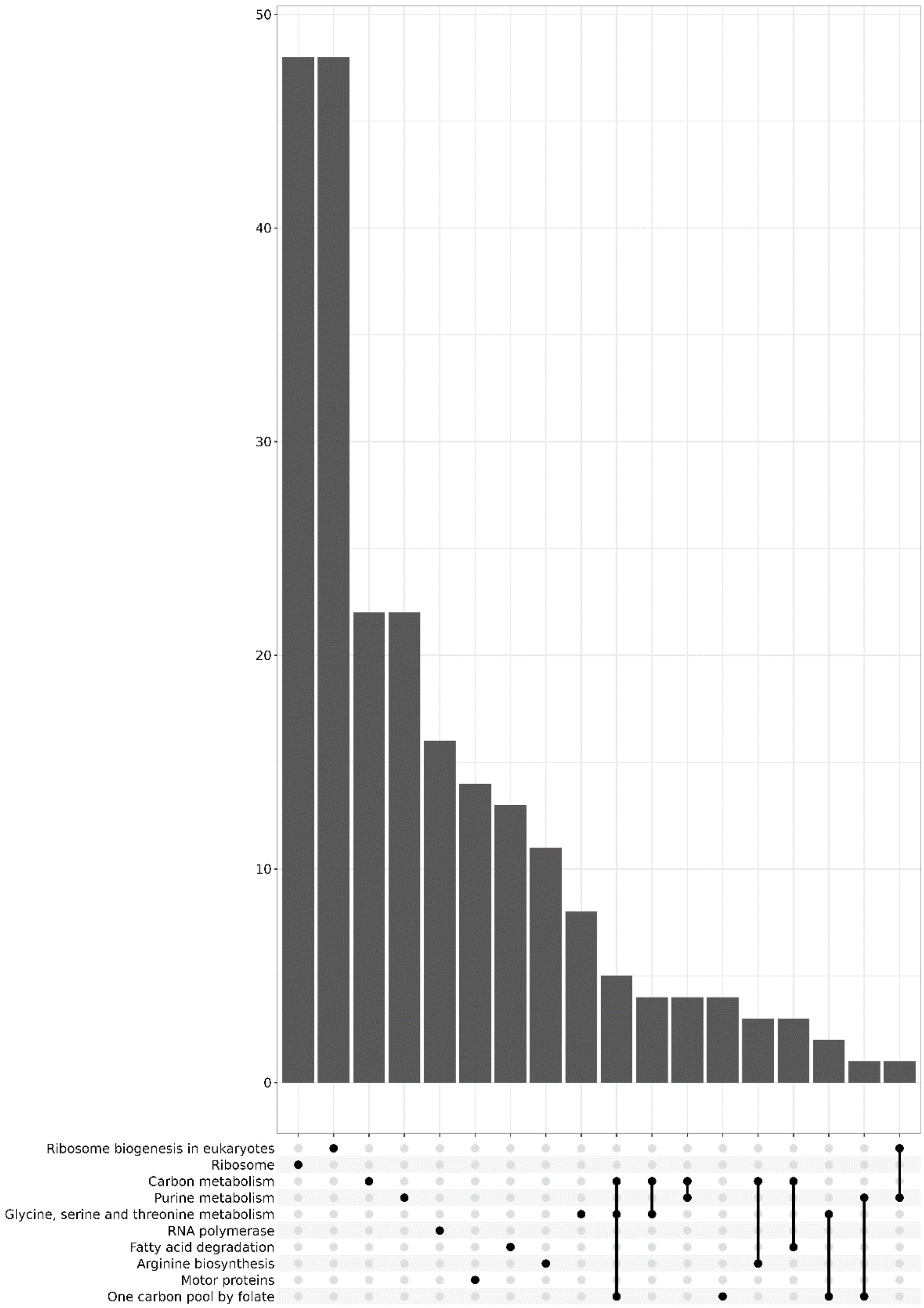

Figure S7. Intersection of KEGG enriched pathways and respective DEGs of miltefosine-treated *Candida tropicalis*. UpSet plot summarizing the overlap of DEGs among the top KEGG-enriched pathways in *C. tropicalis* treated with miltefosine *versus* untreated control in RPMI. Horizontal bars on the left indicate the total number of DEGs assigned to each individual pathway. The matrix of filled dots below indicates specific pathway combinations; connected black dots represent intersections between pathways. Vertical bars above the matrix show the number of DEGs shared by each pathway combination.

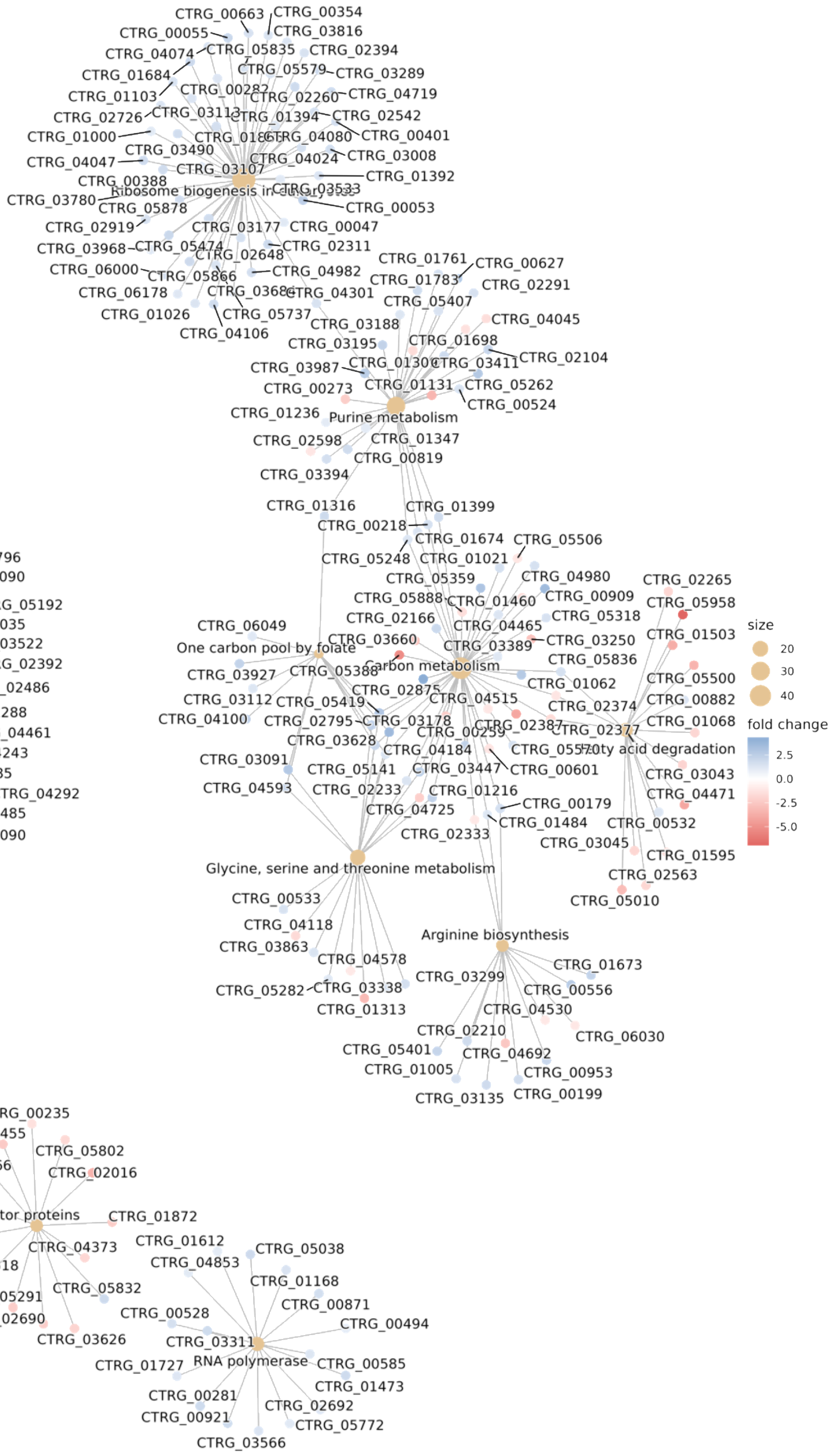

Figure S8. Gene–pathway network of KEGG-enriched terms in miltefosine-treated *Candida tropicalis*. Cnetplot showing the network formed by the most significantly enriched KEGG pathways (large nodes) and their associated differentially expressed genes (small nodes) in *C. tropicalis* treated with miltefosine *versus* untreated control in RPMI. Edges connect each gene to the pathways in which it is annotated, highlighting genes shared by multiple pathways. Node color represents the log_2 fold change in expression (blue, up-regulated genes; red, down-regulated genes), and node size for pathways reflects the number of DEGs mapped to each term.

Supplementary references:

1. de Souza CM, Moralez ATP, Dos Santos MM, Mantovani MS, Furlaneto-Maia L, Furlaneto MC. Deciphering Colonies of Phenotypic Switching-Derived Morphotypes of the Pathogenic Yeast Candida tropicalis. Mycopathologia. 2022 Dec;187(5-6):509-516. doi: 10.1007/s11046-022-00663-4.

2. Silva S, Hooper SJ, Henriques M, Oliveira R, Azeredo J, Williams DW. The role of secreted aspartyl proteinases in Candida tropicalis invasion and damage of oral mucosa. Clin Microbiol Infect. 2011 Feb;17(2):264-72. doi: 10.1111/j.1469-0691.2010.03248.x.

3. Zhang Q, Xu L, Yuan S, Zhou Q, Wang X, Wang L, Hu Z, Yan Y. *NGT1* Is Essential for *N*-Acetylglucosamine-Mediated Filamentous Growth Inhibition and *HXK1* Functions as a Positive Regulator of Filamentous Growth in *Candida tropicalis*. Int J Mol Sci. 2020 Jun 5;21(11):4036. doi: 10.3390/ijms21114036.

4. Oh SH, Isenhower A, Rodriguez-Bobadilla R, Smith B, Jones J, Hubka V, Fields C, Hernandez A, Hoyer LL. Pursuing Advances in DNA Sequencing Technology to Solve a Complex Genomic Jigsaw Puzzle: The Agglutinin-Like Sequence (*ALS*) Genes of *Candida tropicalis*. Front Microbiol. 2021 Jan 20;11:594531. doi: 10.3389/fmicb.2020.594531.

5. Jin L, Cao Z, Wang Q, Wang Y, Wang X, Chen H, Wang H. MDR1 overexpression combined with ERG11 mutations induce high-level fluconazole resistance in Candida tropicalis clinical isolates. BMC Infect Dis. 2018 Apr 10;18(1):162. doi: 10.1186/s12879-018-3082-0.

6. Lin CJ, Wu CY, Yu SJ, Chen YL. Protein kinase A governs growth and virulence in Candida tropicalis. Virulence. 2018 Jan 1;9(1):331-347. doi: 10.1080/21505594.2017.1414132.

7. Maheronnaghsh M, Teimoori A, Dehghan P, Fatahinia M. The evaluation of the overexpression of the ERG-11, MDR-1, CDR-1, and CDR-2 genes in fluconazole-resistant Candida albicans isolated from Ahvazian cancer patients with oral candidiasis. J Clin Lab Anal. 2022 Feb;36(2):e24208. doi: 10.1002/jcla.24208.
